# A fluorescent photoaffinity probe for formyl peptide receptor 1 labelling in living cells†

**DOI:** 10.1039/d2cb00199c

**Published:** 2023-01-13

**Authors:** Devon H. Field, Jack S. White, Stuart L. Warriner, Megan H. Wright

**Affiliations:** a Astbury Centre for Structural Molecular Biology, and the School of Chemistry, University of Leeds, Woodhouse Lane Leeds LS2 9JT UK m.h.wright@leeds.ac.uk

## Abstract

Fluorescent ligands for G-protein coupled receptors (GPCRs) are valuable tools for studying the expression, pharmacology and modulation of these therapeutically important proteins in living cells. Here we report a fluorescent photoaffinity probe for Formyl peptide receptor 1 (FPR1), a critical component of the innate immune response to bacterial infection and a promising target in inflammatory diseases. We demonstrate that the probe binds and covalently crosslinks to FPR1 with good specificity at nanomolar concentrations in living cells and is a useful tool for visualisation and characterisation of this receptor.

## Abstract 2

Through the incorporation of a diazirine photocrosslinking group and a fluorophore, we developed and validated a photoaffinity probe with good selectivity and potent binding to formyl peptide receptor 1 in living cells.

## Graphical abstract

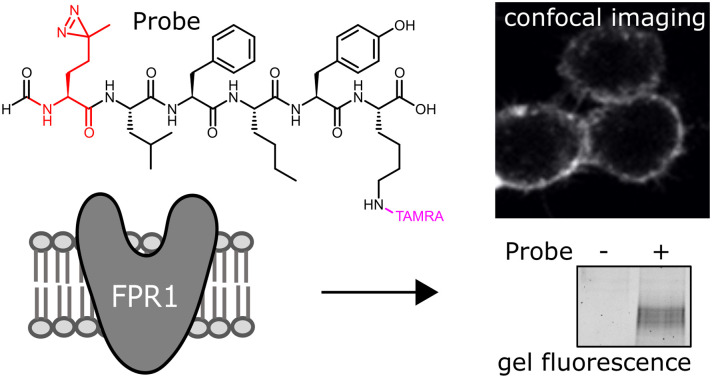

## Introduction

Formyl peptide receptors (FPRs) 1–3 are class A GPCRs that play critical roles in host defence against infection and in inflammation.^1^ They recognise a highly diverse range of endogenous ligands, including bacteria, virus and host-derived peptides, and lipid mediators.^2^ FPR1 is expressed on the surface of leukocytes, where its canonical role is in the regulation of immune responses through recognition of microbe- and damage-associated molecular patterns such as *N*-formyl methionine peptides derived from bacteria and mitochondria.^1,3^ FPR1 has also been reported to be expressed in many different cell types, including a variety of epithelial and cancer cells.^2^ In some cases, evidence for the presence of FPR1 is through transcript detection or immunostaining, and biological relevance or role in these non-canonical settings is often unclear.

FPRs are increasingly recognised for their roles in inflammation,^4^ with therapeutic potential for activation or inhibition, depending on context. For example, FPR1 is required for effective immune recognition of tumour cells during chemotherapy^5^ but its activation during intracerebral haemorrhage exacerbates brain inflammation.^6^ Imaging of FPR1 is of growing interest for diagnostics. For example a fluorescent FPR1 peptide-based probe was recently used to visualise infiltrating neutrophils during abdominal aortic aneurysm in mice,^7^ and imaging has revealed the location of granulomas associated with Tuberculosis infection.^8^

A greater understanding of the fundamental biology of this receptor (*e.g.* dimerisation, trafficking) and its expression and localisation in cells and tissues, would aid in defining when and where FPR1 could be targeted for therapeutic or diagnostic gain. We sought to design here a chemical probe to label FPR1 to irreversibly introduce a fluorophore to this receptor to study its cell biology *in situ*.

## Results and discussion

### Design and synthesis of an FPR1 photoaffinity probe

**Fig. 1:**
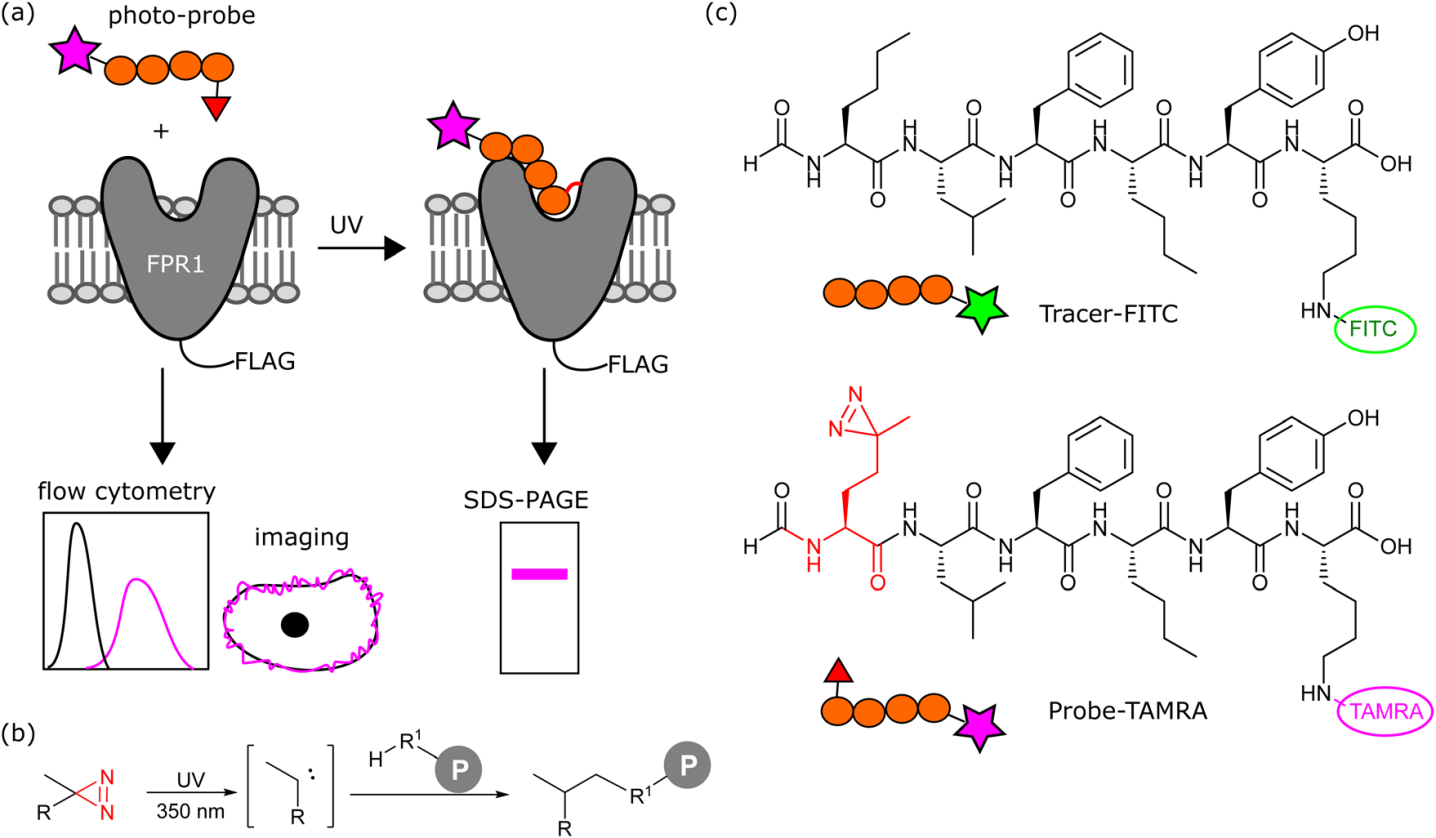
(a) Workflow of photoaffinity labelling of FPR1. A photo-probe is incubated with cells expressing FLAG-tagged FPR1 and reversible binding monitored by flow cytometry and live cell imaging. Upon UV irradiation, the photocrosslinker is activated and a proportion of bound ligand crosslinks to FPR1. Following cell lysis, the crosslinked complex is analysed by reducing SDS-PAGE and in-gel fluorescence. (b) Simplified crosslinking reaction between an alkyl-diazirine probe and a protein (P) *via* a carbene intermediate. (c) Structures of Tracer-FITC and probe-TAMRA.

We aimed to design a chemical probe to specifically, covalently label FPR1 on the surface of cells. Photocrosslinkers have a history of successful use in trapping GPCR-peptide ligand interactions^9^ and a benzophenone-based fluorescent probe has been shown to bind FPR1, act as an agonist and enable crude mapping of the binding site.^10^ However, benzophenone has limited resolution as a photocrosslinker and its bulk and hydrophobicity can enhance non-specific binding.^11,12^ Three recent structures of formyl peptide ligands (fMLF and fMLFII) bound to FPR1 show that the formylated N-terminus reaches deep inside the receptor to a cluster of hydrophobic residues proposed to act as a nexus for activation.^13–15^ We hypothesised that alkyl diazirines, with their small size, rapid irreversible activation and crosslinking, and non-polar properties,^16^ would be ideal crosslinking groups for incorporation into FPR1 ligands (Fig. 1b).

We designed a probe based on the fluorescent tracer formyl-Nle-Leu-Phe-Nle-Tyr-Lys(FITC)-OH (Tracer-FITC, Fig. 1c), which has a reported K_D_ of ∼3 nM and has been widely used in flow cytometry assays for FPR1.^17^ We prepared Tracer-FITC by solid phase peptide synthesis: the N-terminus was formylated *via* an optimised procedure using *p*-nitrophenyl formate^18^ and the lysine side chain was protected with an orthogonal protecting group (ivDde) that was removed using 4% hydrazine prior to coupling of the FITC fluorophore. To create a probe and incorporate a crosslinking group with minimal changes to this structure, we replaced the N-terminal norleucine residue with photo-methionine.^19^ Probe-TAMRA (Fig. 1c) was prepared *via* SPPS with the C-terminal fluorescein replaced with 5(6)-TAMRA to enable orthogonal visualisation of the two reagents in assays. A TAMRA version of the tracer has previously been used to visualise FPR internalisation in cells,^20^ suggesting that the nature of the fluorophore is not critical to receptor binding. Probe-TAMRA was prepared in an analogous procedure to Tracer-FITC and purified by preparative HPLC in 98% purity and 16% overall yield.

### Evaluation of probe-TAMRA binding to FPR1

**Fig. 2:**
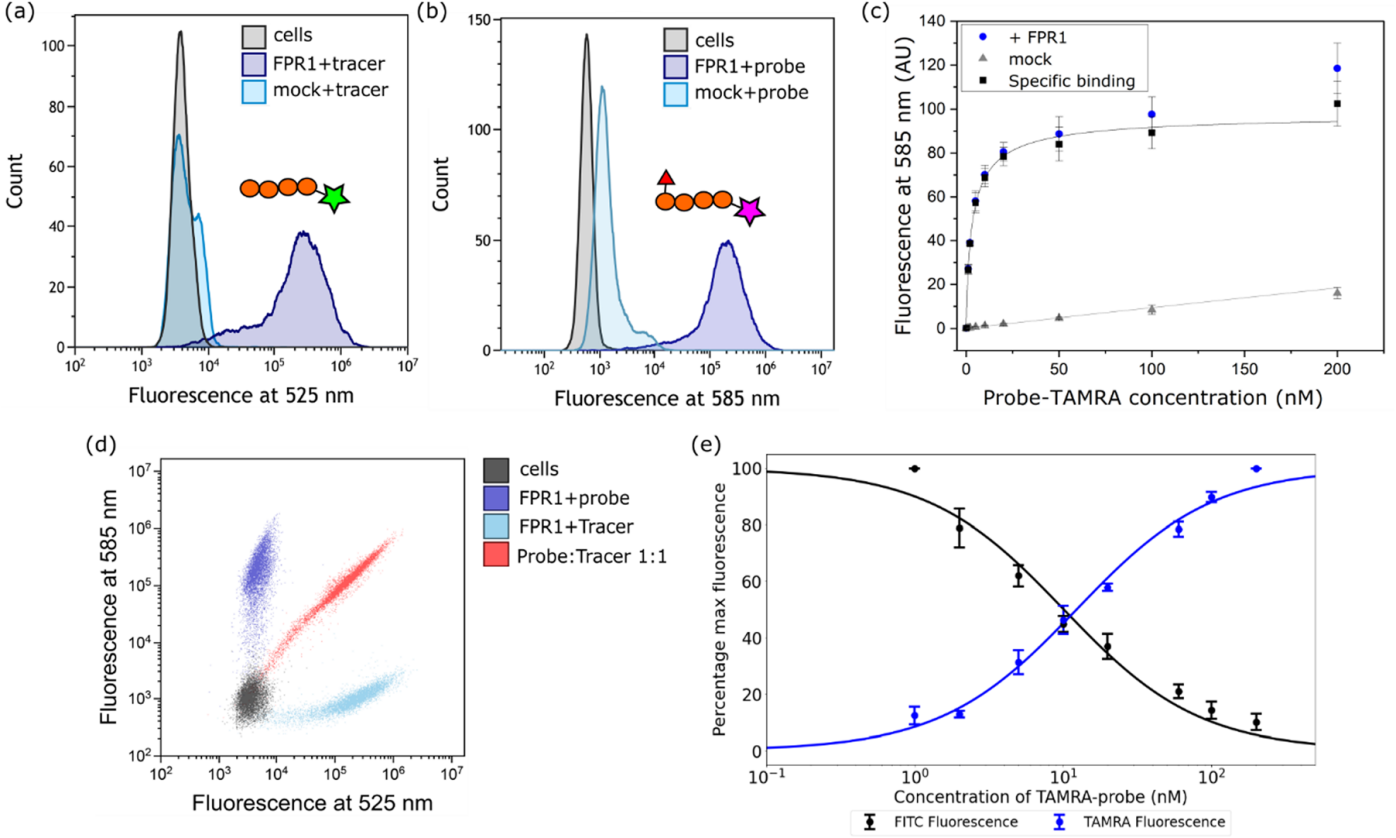
Evaluation of probe-TAMRA as a ligand for FPR1 by flow cytometry. Histograms of FPR1-transfected (+FPR1) or mock-transfected (mock) HEK293T cells incubated with Tracer-FITC (a) or probe-TAMRA (b) each at 10 nM at the indicated wavelengths. 10 000 cells measured per sample. (c) Plot of mean fluorescence intensity determined from flow cytometry analysis following incubation with increasing concentrations of probe-TAMRA with transfected (blue) or mock transfected (grey) cells. Data from 3 biological replicates; error bars represent standard error of the mean. Black curve is subtraction of background binding (grey curve) from blue curve and represents specific binding. (d) Dot plot from incubation of transfected cells with Tracer-FITC alone (cyan), probe-TAMRA alone (blue), or co-incubation of probe and tracer (red). All peptides at 10 nM. (e) Plot of percentage maximum fluorescence at 525 nm (FITC) and 585 nm (TAMRA) wavelengths from a flow cytometry competition experiment in which Tracer-FITC was held constant at a concentration of 10 nM and the concentration of probe-TAMRA increased from 0–200 nM. Data from 3 biological replicates; error bars represent standard error of the mean.

Next, we established a model system for evaluating our probe in cells. HEK293T cells were transiently transfected with C-terminally FLAG-tagged FPR1, yielding a diffuse band by Western blot that resolved to give a tighter band of the expected molecular weight upon deglycosylation with PNGaseF (ESI,† Fig. S1). Flow cytometry analysis of HEK293T cells incubated with 10 nM Tracer-FITC showed a clear shift to a population of fluorescent cells with minimal background binding in mock-transfected cells (Fig. 2a). We were pleased to observe that probe-TAMRA gave a similar increase in fluorescence in flow cytometry analysis at the relevant wavelength (Fig. 2b) and titration of probe from 0–200 nM showed low background binding that did not exceed 15% of total binding (Fig. 2c). Following background subtraction, a *K*_D_ of 3.5 ± 0.9 nM can be calculated for probe-TAMRA.

The different fluorophores in the tracer and probe should allow both to be monitored during competition experiments. Incubation of FPR1-expressing cells with Tracer-FITC or probe-TAMRA showed clear populations of cells at the appropriate wavelengths with little crosstalk between the fluorophores (blue and cyan dots in Fig. 2d). Co-incubation of the tracer and probe showed clear competition (red dots, Fig. 2d). We then performed a titration, keeping the concentration of Tracer-FITC constant at 10 nM and increasing the concentration of probe-TAMRA up to 200 nM. This gave the expected decrease in FITC signal with a concomitant increase in TAMRA signal (ESI,† Fig. S2) and a plot of the percentage of maximum fluorescence for each showed concentration-dependent competition (Fig. 2e). These data indicate that the probe and tracer most likely bind the same site. The similar affinity of Tracer-FITC and probe-TAMRA we observed is in keeping with previous data showing that formyl-Nle-Leu-Phe-Nle-Tyr-Lys(TAMRA)-OH, like Tracer-FITC, also exhibits low nM binding to FPR1, suggesting that the nature of the fluorophore itself is not important for binding.^20^ No structural information is available for fluorescent peptide FPR1 ligands but based on the binding of a 5-residue peptide,^13^ these 6-residue fluorophore-linked probes might be expected to bind with the fluorophore close to the pocket entrance.

We then attempted competition experiments with known ligands. We could only observe competition of probe-TAMRA binding with the canonical FPR1 ligand fMLF at concentrations greater than 5 μM (ESI,† Fig. S2), which is surprising given that the *K*_D_ of this tripeptide is reported in the nM range.^21^ However, a survey of the literature showed that fMLF is often used at micromolar concentrations to achieve competition and that this is highly dependent on the competing fluorescent ligand.^22–24^ Pleasingly, known antagonist Boc-MLF could outcompete probe-TAMRA at concentrations of 10–50 μM, consistent with the literature (ESI,† Fig. S2).^25^ Together these data suggest highly specific binding of probe-TAMRA to FPR1.

### Probe-TAMRA for visualising FPR1 internalisation in live cells

**Fig. 3:**
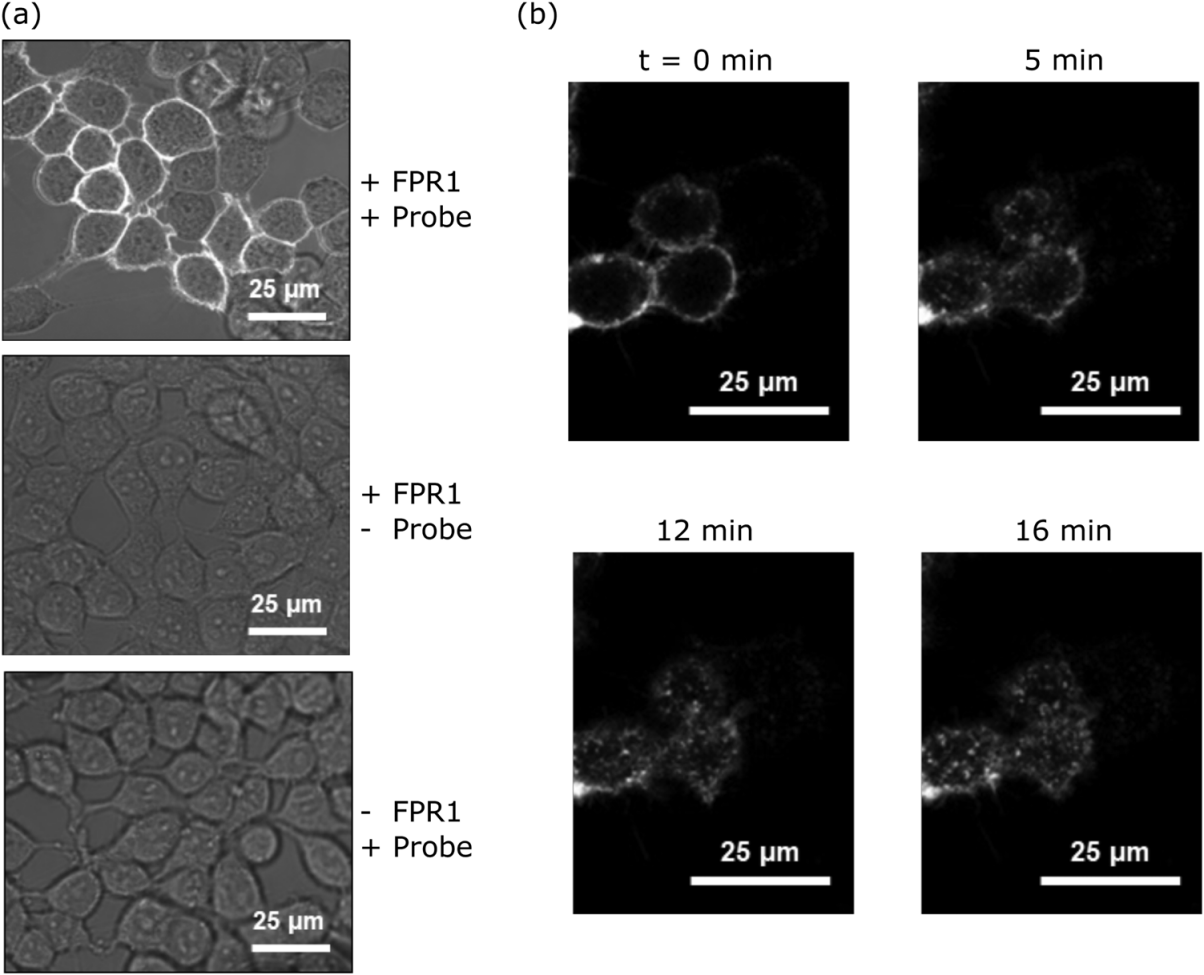
Imaging of cells incubated with probe-TAMRA. (a) Confocal microscopy images of HEK293T cells incubated with probe-TAMRA (10 nM) on ice for 30 min; images are the overlay of brightfield and fluorescence at 561 nm. (b) Time course of internalisation of probe-FPR1 complex; HEK293T cells transfected overnight at 37 °C, incubated with 10 nM probe-TAMRA at 0 °C for 30 min; fluorescence imaged at 561 nm with confocal microscope for 16 min after cells were warmed to 37 °C (at *t* = 0 min).

Upon agonist binding, FPR1 internalises following activation of G protein subunits, resulting in receptor desensitisation.^20^ To determine whether probe-TAMRA exhibited similar behaviour and to assess its potential for FPR1 imaging, we incubated HEK293T cells transfected with FPR1 with 10 nM probe-TAMRA in glass-well culture dishes on ice for 30 minutes and imaged these by confocal microscopy following washing. Clear fluorescent signal was observed on the membrane of transfected cells with low background in non-transfected controls (Fig. 3a). Upon heating to 37 °C, fluorescent material was observed to transfer into the cytosol (Fig. 3b), suggesting receptor internalisation similar to observations made with peptide formyl-Nle-Leu-Phe-Nle-Tyr-Lys(TAMRA)-OH in previous studies.^20^ This shows that probe-TAMRA binds to FPR1 on the surface and likely acts as an agonist to trigger receptor internalisation.

### Probe-TAMRA can crosslink FPR1 with high selectivity in living cells

**Fig. 4:**
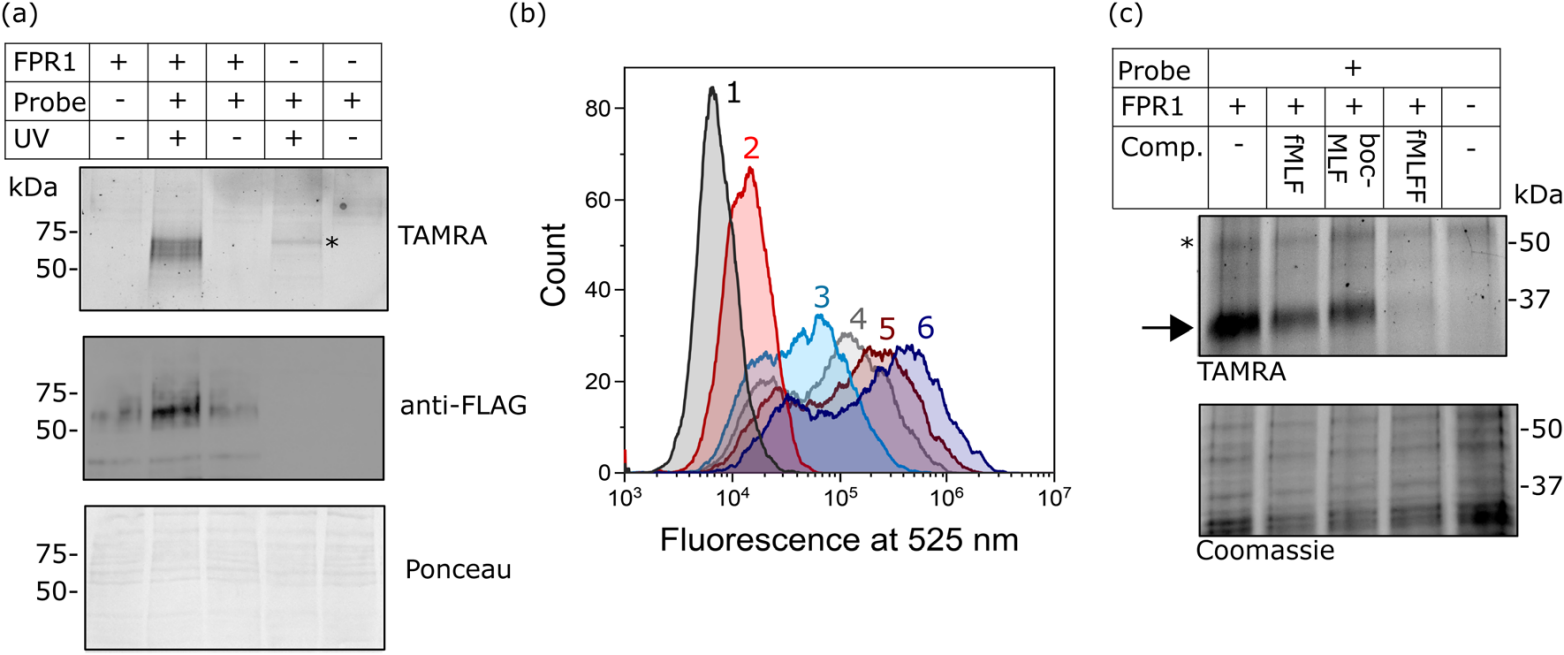
Analysis of the crosslinking of FPR1 to probe-TAMRA. (a) UV-dependent crosslinking analysed by gel and western blot. HEK293T cells transfected with FPR1 or mock transfected were suspended, incubated with probe-TAMRA (10 nM) and irradiated with UV light (365 nm) as indicated. Following cell lysis, samples were separated by SDS-PAGE and analysed by in-gel fluorescence (550 nm emission) and anti-FLAG western blot. Ponceau staining was used to assess protein loading. Full gels and blots in ESI,† Fig. S6. (b) Flow cytometry histograms at 525 nm (FITC fluorescence) of FPR1-transfected HEK293T cells incubated variously, as indicated, with: Tracer-FITC alone (6, dark blue), probe-TAMRA alone (2, red), in combination (3, cyan), or in sequence with photoirradiation (4, 5). For the latter, cells were first incubated with probe-TAMRA for 30 min, irradiated with 365 nm light for 30 s, washed, incubated with Tracer-FITC for 30 min and analysed (5, dark red), or with the inclusion of an addition incubation and irradiation step (4, grey). Probes were used at a concentration of 10 nM. Samples were washed twice before analysis and 10,000 cells were measured per sample. (c) Gel-based analysis of competition of probe-TAMRA labelling with known FPR1 ligands fMLF, BocMLF and fMLFF. HEK293T cells transfected with FPR1 or mock transfected were suspended, lysed by sonication and membrane fraction isolated by centrifugation. Aliquots were co-incubated with probe-TAMRA (50 nM) and competitor ligand (fMLF: 5 μM; BocMLF: 20 μM; fMLFF: 5 μM) or DMSO control for 30 min, washed, resuspended in PBS and irradiated with UV light (365 nm) for 30 s. Proteins were deglycosylated with PNGaseF, separated by SDS-PAGE and analysed by in-gel fluorescence (550nm emission). The gel was Coomassie stained to provide evidence of equal protein loading.

Finally, we investigated whether probe-TAMRA could covalently crosslink to FPR1 in cells. We used a bespoke UV LED device that can achieve highly efficient crosslinking in seconds, minimising sample heating.^26^ Transfected HEK293T cells were suspended with the probe, irradiated at 365 nm in this device for 30 s as previously optimised,^26^ lysed and analysed by SDS-PAGE. Excitingly, we saw a diffuse fluorescent band at ∼60 kDa in the presence of probe and only upon UV irradiation and receptor expression (Fig. 4a). A weak signal at slightly higher molecular weight was detected in the non-transfected sample, indicating crosslinking to an additional protein. This off-target band is not observable in the +FPR1 lane, likely due to the intensity of the FPR1 band. There is a small amount of FPR1-independent binding in flow cytometry data (Fig. 2a) so it is possible that this band corresponds to another protein that also binds the probe.

Crosslinking efficiency appeared similar at room temperature and 4 °C, and tight binding was evident from the fact that the crosslinking signal did not diminish when cells were washed before irradiation (ESI,† Fig. S3). This is consistent with the long-residence time of formyl-peptide agonist ligands at FPR1.^27,28^

To further confirm that the fluorescent species was FPR1, we performed deglycosylation of the sample, which led to the expected decrease in molecular weight of the fluorescent and FLAG-tagged bands and a sharpening of the band as the heterogeneous glycans are removed (ESI,† Fig. S4). We could also pull-down of FPR1 *via* the FLAG-tag, further confirming a covalent probe-protein complex. The off-target band is observed in both experiments after deglycosylation (unaffected by PNGaseF) and as a fluorescent band that is not enriched by FLAG pull-down (ESI,† Fig. S4), as expected.

We also explored whether covalent crosslinking could be detected *via* a competition flow cytometry experiment. As before, incubation of HEK293T cells expressing FPR1 with Tracer-FITC gave rise to a population of fluorescent cells, which decreased in intensity upon co-incubation with probe-TAMRA (Fig. 4b). Sequential treatment of cells with probe-TAMRA, irradiation with UV light, washing, and incubation with Tracer-FITC gave cells with decreased fluorescence (Fig. 4b); a further decrease in signal was seen when a second probe-TAMRA incubation and irradiation step was included before Tracer incubation. A concomitant increase in fluorescent FPR1 labelling was seen on a gel with repeated irradiations (ESI,† Fig. S5). Following background subtraction of the flow cytometry data, a drop in fluorescence intensity of 23% can be calculated for a single round of irradiation, suggesting that around a quarter of available FPR1 sites are crosslinked. Crosslinking yields for alkyl diazirines can be low, in part because they can form less reactive diazo species when irradiated in addition to the desired carbenes.^29^ Our data show that we can detect the irreversible removal of FPR1 binding sites from the cell surface using our photoaffinity probe, and increase crosslinking yield by multiple rounds of probe incubation and irradiation.

Finally, we investigated whether probe-TAMRA combined with in-gel fluorescence analysis could be used to detect binding of competitor ligands to FPR1. To increase throughput for this assay, we first showed that probe-TAMRA could label FPR1 in lysate, which can be easily stored in frozen aliquots and thawed for experiments. FPR1-transfected cells were harvested, lysed by sonication and aliquots of lysate incubated with probe-TAMRA, followed by irradiation and gel-based analysis. Signal-to-noise was slightly lower than for cell-based labelling, requiring an increase to 50 nM probe for visualisation, but probe labelling of FPR1 was clearly detectable (ESI,† Fig. S7). For competition experiments, we co-incubated crude membrane fractions of lysate with probe-TAMRA and various known ligands: fMLF and BocMLF were used at 5 and 20 μM respectively, concentrations at which clear competitive binding was observed by flow cytometry (ESI,† Fig. S2), and a more potent reported ligand, fMLFF,^30^ was also included (also at 5 μM). Membrane fractions were pelleted, resuspended in PBS and irradiated as before, then samples deglycosylated to reduce the molecular weight of FPR1, sharpening the diffuse band and avoiding any interference from the off-target labelled protein. Clear competition is visible for all ligands and this is selective for FPR1 (indicated by the arrow on the gel in Fig. 4c). The off-target band is unaffected (highlighted with a * in Fig. 4c), suggesting that it may be an off-target of the probe rather than a true binder of formyl peptide ligands. fMLF and BocMLF gave incomplete inhibition of labelling at these concentrations, consistent with the flow cytometry data (ESI,† Fig. S7), whereas fMLFF gave complete inhibition.

Together these data demonstrate that probe-TAMRA is a highly potent and selective tool for covalently labelling FPR1 in living cells and for assaying ligand engagement of this receptor.

## Conclusions

Here we report the development of a fluorescent photoaffinity probe for FPR1. Through extensive characterisation we demonstrate that probe-TAMRA binds to the receptor with low nM affinity and in competition with known ligands, acts as an agonist to trigger receptor internalisation, and shows crosslinking with good specificity to FPR1 in cells.

Approximately 30% of drugs target GPCRs^31^ and photoaffinity probes have historically been key tools for studying ligand binding sites, typically using cell membrane fractions and radioactivity for detection.^9^ A challenge for live cell labelling and detection is the very low expression levels of GPCRs, but a handful of recent studies have built on advances in chemical biology (*e.g.* improved synthetic routes to diazirines and better understanding of their photochemistry^32^) to report fluorescent chemical probes and applied them to photoaffinity label GPCRs in living cells.^33–35^ Here we present probe-TAMRA as a tool for investigating the fundamental biology of FPR1 and its viability as a drug target in inflammatory diseases. In the future we aim to apply probe-TAMRA to confirm the presence of FPR1 in endogenous settings, to visualise protein trafficking, and to understand the oligomerisation state of this receptor on the cell surface, as there is evidence that interconverting populations of large and small oligomers play a role in signal transduction.^36^ The photocrosslinking group incorporated into the probe also provides a means of determining the target selectivity of formylpeptides and probes through gel-based analysis; the off-target band, whose identity is unknown at present, that we observe on gel may be a feature of formyl-peptide based tools and warrants further exploration, for example by incorporating an enrichment step and mass spectrometry analysis to detect other binders of the probe. The crosslinker-enabled gel-based approach also provides a means of evaluating ligand binding without relying on flow cytometry, and may prove useful for assessing the on-target mode of action of FPR1-directed modulators in the future.

## Data availability

The data associated with this paper are openly available from the University of Leeds Data Repository https://doi.org/10.5518/1226.

## Author contributions

DHF, JSW and MHW designed and performed experiments. DHF and MHW analysed data. MHW and SLW conceived, designed and supervised the research. MHW and DHF wrote the manuscript with input from all authors.

## Conflicts of interest

There are no conflicts to declare.

## Supplementary Material

CB-004-D2CB00199C-s001
