# Supplementary material for: A fluorescent photoaffinity probe for formyl peptide receptor 1 labelling in living cells
Source: RSC Chem Biol. 2023 Jan 13;4(3):216–22. doi: 10.1039/d2cb00199c (PMC9994102; doi:10.1039/d2cb00199c)
Supplement: CB-004-D2CB00199C-s001 [file CB-004-D2CB00199C-s001.pdf]

Electronic Supplementary Material (ESI) for RSC Chemical Biology.
This journal is © The Royal Society of Chemistry 2023

Supplementary Information

A fluorescent photoaffinity probe for formyl peptide receptor 1 labelling in living cells

Devon H. Field, Jack S. White, Stuart L. Warriner and Megan H. Wright*

Contents

1 Synthetic Chemistry 2
1.1 General reagents and equipment 2
1.2 Synthesis of Fmoc-photo-Met 3
1.3 Synthesis of Tracer-FITC 7
1.4 Synthesis of Probe-TAMRA 9
2 Biological and biochemical methods 11
2.1 General Methods and Equipment 11
2.2 Media and buffers 11
2.2.1 Growth media 11
2.2.2 Buffers for protein and DNA analysis 11
2.3 Bacterial Transformation of FPR1 gene cDNA ORF 12
2.3.1 Transformation of *E. coli* Cells 12
2.3.2 Plasmid isolation and quality control 12
2.4 Mammalian cell culture, photocrosslinking and lysis 13
2.4.1 Transient Transfection 13
2.4.2 Probe Incubation, crosslinking and lysis on suspended cells 13
2.4.3 Probe incubation and crosslinking experiments on lysates 13
2.5 Gel- and Western blot analysis of samples 13
2.6 Deglycosylation of FPR1 14
2.7 Anti-FLAG Pull-Down 14
2.8 Flow Cytometry 14
2.9 Confocal Microscopy 15
3 Supplementary Figures 16
4 Appendix: NMR spectra for compounds 1-7 (PhotoMet synthesis) 22
5 References 26

1 Synthetic Chemistry

1.1 General reagents and equipment

All glassware used was dried in an oven prior to the reaction. Unless otherwise stated all commercial reagents were used as received and reactions were performed in an inert atmosphere using N_2. All reactions were monitored by analytical thin layer chromatography using aluminium TLC plates coated with silica gel $60F_{254}$ purchased from Merck. TLC plates were visualised by UV light (λ=254 nm) and an aqueous potassium permanganate dip.

Flash column chromatography was performed using silica gel (60 Å, 40 – 63 micron) purchased from Merck.

^{1}H NMR and ^{13}C NMR spectra were recorded using a Bruker 400 ultra shield spectrometer (400 MHz) in $CDCl_3$ (reference of 7.26 for ^{1}H NMR and 77.2 for ^{13}C NMR) and MeOD (reference of 4.78 and 3.31 for ^{1}H NMR, and 49.15 for ^{13}C NMR). All chemical shifts are expressed in parts per million downfield from tetramethylsilane. Peak splittings are noted as singlet (s), doublet (d), triplet (t), quartet (q), pentet (p) and multiple (m) and combinations of the stated J coupling constants are recorded to the nearest 0.5 Hz.

Low resolution electrospray (ES+) ionisation mass spectra were obtained on a Bruker Amazon mass spectrometer. High resolution ES+ mass spectra were obtained on a Bruker MaXis Impact mass spectrometer.

For solid phase peptide synthesis, all amino acids and other reagents were purchased from Novabiochem and Sigma Aldrich and were used without further purification.

Fritted polypropylene tubes (10 mL) were purchased from Biotage and used as the vessels for peptide synthesis. Dissolution of reagents and peptides was achieved by agitation through the use of a Stuart rotator. 2-Chlorotrityl chloride resin (loading 1.33 mmol/g) was used as the stationary phase for peptide synthesis.

Analysis of peptides during SPPS was performed using a Thermo Ultimate 3000 UHPLC, Bruker Amazon Speed ion trap mass spec with a Phenomenex Aeris Peptide XB C18 column (100 x 2.1mm, 2.6um particle size). Gradient from 0.1% TFA/ 2% MeCN (v/v) in water to 0.1% TFA/ 98% MeCN (v/v) in water with a flow rate of 0.85ml/min.

Analysis of final peptides was performed using an Agilent 1290 Infinity with Diode Array Detection with an Ascentis Peptide ES C18 column (100 x 2.1mm, 2.7um particle size). Gradient from 0.1% TFA/ 5% MeCN (v/v) in water to 0.1% TFA/ 95% MeCN (v/v) in water with a flow rate of 0.5ml/min.

1.2 Synthesis of Fmoc-photo-Met

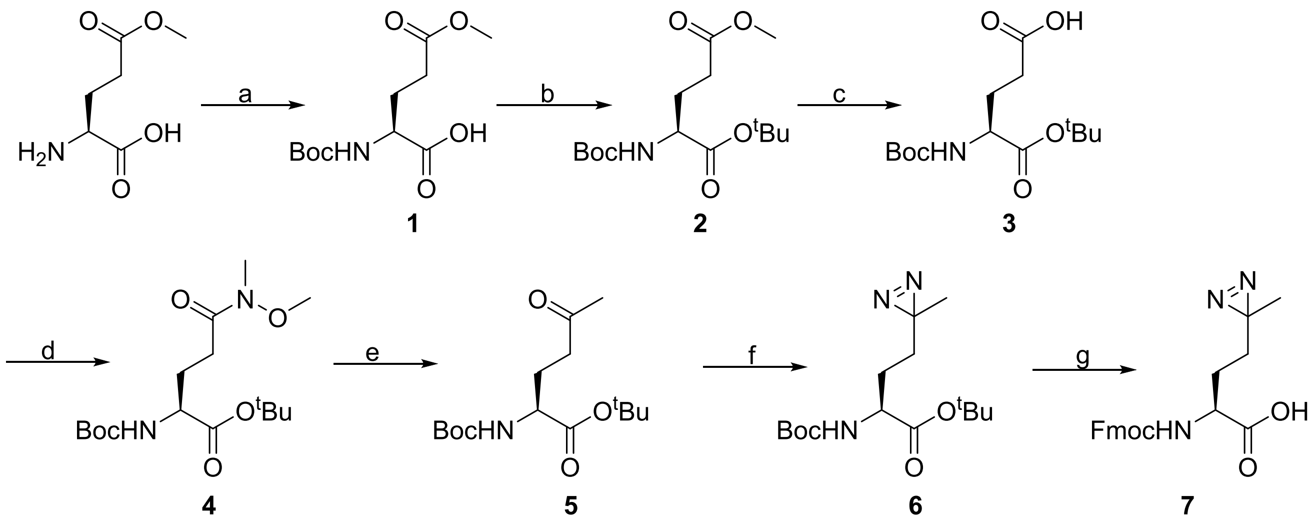

Reagents and conditions: (a) Boc_2O, $NaHCO_3$, dioxane/water (2:1), 0 °C, **76%**; (b) DCC, DMAP, ^{t}BuOH, DCM, 0 °C, **76%**; (c) LiOH(aq), THF, **96%**, (d) i) *N*-methylmorpholine, isobutyl chloroformate, DCM, 0 °C; ii) N,O-dimethylhydroxylamine hydrochloride, DCM, 0 °C, **84%**; (e) MeMgBr, toluene, −78 °C, **64%**; (f) i) NH_3, hydroxylamine-O-sulfonic acid, –10 °C; ii) MeOH, Et_3N, iodine, 0 °C, **94%**; (g) i) THF:4M HCl (1:1); ii) FmocOSu, $NaHCO_3$, water:dioxane (1:1), **73%.**

Scheme S1: Synthesis of Fmoc-photo-Met

(S)-2-((*tert*-butoxycarbonyl)amino)-5-methoxy-5-oxopentanoic acid (Boc-Glu(OMe)-OH), 1

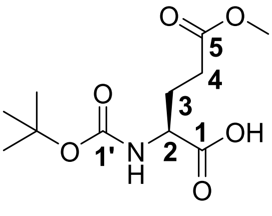

1

H-Glu(OMe)-OH (10.0 g, 50.6 mmol) was dissolved in dioxane/water (2:1, 150 mL) and cooled to 0 °C. Boc_2O (13.2 g, 60.7 mmol) and $NaHCO_3$ (10.6 g, 127 mmol) were added and the reaction mixture was warmed up to room temperature, before being stirred for 12 hr. The dioxane was removed under reduced pressure and the aqueous solution was washed with diethyl ether (50 mL). 1M HCl was added to adjust the pH to 3 and the resulting solution was extracted with ethyl acetate (2 × 50 mL). The organic solution was washed with water (50 mL) and brine (50 mL), and dried over anhydrous Na_2SO_4. Removal of the solvent under reduced pressure yielded the product **1** as a pale yellow oil (10.0 g, 38.3 mmol, 76%).

^{1}H NMR (400 MHz, $CDCl_3$) δ 5.22 (1H, d, *J* = 8.0 Hz, NH), 4.40-4.29 (1H, m, C-2), 3.68 (3H, s, Me), 2.53-2.38 (2H, m, C-4), 2.27-2.19 (1H, m, C-3), 2.05-1.96 (1H, m, C-3), 1.43 (9H, s, ^{t}Bu). **HRMS** (ES+) m/z calculated for $C_{11}H_{19}NO_6Na$ $[M+Na]^+$ 284.1105, found 284.1104; $C_{11}H_{19}NO_6K$ $[M+K]^+$ 300.0844, found 300.0844; **R_f** 0.33 (40% ethyl acetate in pentane).[1, 2]

(S)-5-(*tert*-butoxy)-4-((*tert*-butoxycarbonyl)amino)-5-oxopentanoic acid (Boc- Glu(OMe)-O*t*-Bu), 2

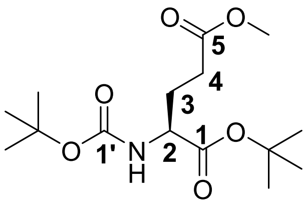

2

DCC (9.47 g, 45.9 mmol), DMAP (469 mg, 3.84 mmol) and ^{t}BuOH (36.6 mL, 383 mmol) were added to ice-cold DCM (150 mL). Boc- Glu(OMe)-OH (10.03 g, 38.39 mmol) was dissolved in DCM (100 mL) and added dropwise to the ice-cold mixture. The reaction solution was stirred for one h at 0 °C and allowed to warm to room temperature over 12 h with stirring. The DCM was removed under reduced pressure and the resulting white solid was re-dissolved in ethyl acetate (100 mL). The suspension was filtered through celite to remove DCU and the filtrate washed with 0.1 M HCl (100 mL), saturated sodium $NaHCO_3$ (100 mL) and brine (100 mL). The organic solution was dried over anhydrous Na_2SO_4 and concentrated under reduced pressure. The crude product was purified using column chromatography (10% ethyl acetate in pentane) to yield the product **2** as a white solid (9.30 g, 29.3 mmol, 76%).

^{1}H NMR (400 MHz, $CDCl_3$) δ 5.07 (1H, d, *J* = 8.5 Hz, NH), 4.19-4.09 (1H, m, C-2), 3.67 (3H, s, Me), 2.46-2.31 (2H, m, C-4), 2.19-2.10 (1H, m, C-3), 1.95-1.86 (1H, m, C-3), 1.46 (9H, s, ^{t}Bu), 1.43 (9H, s, ^{t}Bu); **HRMS** (ES+) m/z calculated for $C_{15}H_{27}NO_6Na$ $[M+Na]^+$ 340.1731, found 340.1741; **R_f** 0.30 (30% ethyl acetate in pentane).[1, 2]

(S)-5-(*tert*-butoxy)-4-((*tert*-butoxycarbonyl)amino)-5-oxopentanoic acid (Boc- Glu(OH)-O*t*-Bu), 3

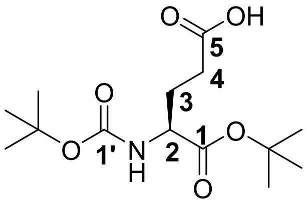

3

A 1M LiOH solution (15.8 mL, 15.8 mmol) was added dropwise to a solution of Boc-Glu(OH)-O*t*-Bu (2.5 g, 7.9 mmol) in THF (50 mL) over 30 min. The reaction mixture was left to stir for 2 h before being cooled to 0 °C. 0.1M HCL was added dropwise to the solution until the pH was 5. The organic layer was extracted with ethyl acetate (2 × 50 mL), washed with brine (50 mL) and dried over anhydrous Na_2SO_4. Concentrating under reduced pressure yielded the product **3** as a white solid (2.3 g, 7.6 mmol, 96%).

^{1}H NMR (400 MHz, $CDCl_3$) δ 5.16 (1H, d, *J* = 8.0 Hz, NH), 4.29-4.07 (1H, m, C-2), 2.53-2.37 (2H, m, C-4), 2.23-2.09 (1H, m, C-3), 1.99-1.84 (1H, m, C-3), 1.47 (9H, s, ^{t}Bu), 1.44 (9H, s, ^{t}Bu); **HRMS** (ES+) m/z calculated for $C_{14}H_{25}NO_6Na$ $[M+Na]^+$ 326.1574, found 326.1580; **R_f** 0.44 (30% ethyl acetate in pentane, 1% acetic acid).[2, 3]

(S)-*tert*-butyl 2-((*tert*-butoxycarbonyl)amino)-5-(methoxy(methyl)amino)-5- oxopentanoate, 4

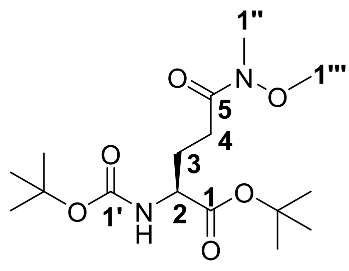

4

Boc- Glu(OH)-O*t*-Bu (300 mg, 0.99 mmol) dissolved in DCM (25 mL) and isobutyl chloroformate (0.17 mL, 1.3 mmol) were added to *N*-methylmorpholine (0.27 mL, 2.5 mmol) at 0 °C. The reaction mixture was stirred for one h at 0 °C before *N*,*O*-dimethylhydroxylamine hydrochloride (116 mg, 1.19 mmol) was added portion-wise. The reaction mixture was stirred for a further 16 h at room temperature and subsequently quenched with 0.1 M HCl (10 mL). The organics were extracted with DCM (3 × 20 mL), washed with brine (30 mL) and dried over anhydrous Na_2SO_4. The crude product was concentrated under reduced pressure and purified via column chromatography (30% ethyl acetate in pentane) to furnish the product **4** as a colourless oil which solidified upon standing (290 mg, 0.84 mmol, 84%).

^{1}H NMR (400 MHz, CDCl$_3$) δ 5.19 (1H, d, *J* = 8.5 Hz, NH), 4.27-4.16 (1H, m, C-2), 3.69 (3H, s, C-1'''), 3.20 (3H, s, C-1''), 2.63-2.44 (2H, m, C-4), 2.23-2.12 (1H, m, C-3), 1.99-1.90 (1H, m, C-3), 1.49 (9H, s, ^{t}Bu), 1.46 (9H, s, ^{t}Bu); **HRMS** (ES+) m/z calculated for $C_{16}H_{30}N_2O_6Na$ $[M+Na]^+$ 369.1996, found 369.1991; $C_{16}H_{30}N_2O_6K$ $[M+K]^+$ 385.1735, found 385.1726; $C_{32}H_{60}N_4O_{12}Na$ $[2M+Na]^+$ 715.4100, found 715.4098; **R$_f$** 0.29 (60% ethyl acetate in pentane).[2, 3]

(S)-*tert*-butyl-2-((*tert*-butoxycarbonyl)amino)-5-oxohexanoate, 5

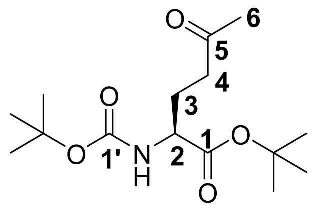

5

(S)-*tert*-butyl-2-((*tert*-butoxycarbonyl)amino)-5-(methoxy(methyl)amino)-5-oxopentanoate (1.07 g, 3.08 mmol) was dissolved in toluene (50 mL) and cooled to −78 °C. Methylmagnesium bromide solution 3.0 M (2.1 mL, 6.34 mmol) was added dropwise over 30 min, after which the solution was warmed to −5 °C and stirred for three hr. The reaction was quenched with 0.1 M HCl (20 mL) and the organic solution extracted with ethyl acetate (3 × 20 mL). The solution was dried over anhydrous Na_2SO_4 and concentrated under reduced pressure. The crude product was purified via column chromatography (10% ethyl acetate in hexane) to furnish the product **5** as a colourless oil (0.56 g, 1.84 mmol, 64%).

^{1}H NMR (400 MHz, CDCl$_3$) δ 5.05 (1H, d, *J* = 6.0 Hz, NH), 4.19-4.09 (1H, m, C-2), 2.63-2.43 (2H, m, C-4), 2.15 (3H, s, Me), 2.13-2.05 (1H, m, C-3), 1.90-1.78 (1H, m, C-3), 1.49 (9H, s, ^{t}Bu), 1.44 (9H, s, ^{t}Bu); **HRMS** (ES+) m/z calculated for $C_{15}H_{27}NO_5Na$ $[M+Na]^+$ 324.1781, found 324.1785; $C_{15}H_{27}NO_5K$ $[M+K]^+$ 340.1521, found 340.1517; $C_{30}H_{54}N_2O_{10}Na$ $[2M+Na]^+$ 625.3671, found 625.3670; **R$_f$** 0.44 (60% ethyl acetate in hexane).[2, 3]

(S)-tert-butyl 2-((tert-butoxycarbonyl)amino)-4-(3-methyl-3H-diazirin-3-yl)butanoate, 6

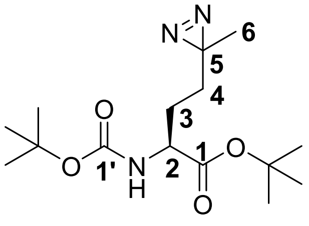

6

(S)-*tert*-butyl 2-((*tert*-butoxycarbonyl)amino)-5-oxohexanoate (1.0 g, 3.3 mmol) was dissolved in liquid ammonia (30 mL) by the condensing of ammonia gas with a dry ice-acetone condenser. The solution was stirred for 5 h with the condenser kept at −78 °C. Hydroxylamine-*O*-sulfonic acid (441 mg, 3.90 mmol) was dissolved in dry MeOH (10 mL) and added dropwise to the reaction solution. The mixture was stirred overnight, being allowed to warm to room temperature. Nitrogen was subsequently blown through the reaction for one h to remove any ammonia. The solution was then filtered, washed with MeOH (20 mL) and concentrated under reduced pressure. The resulting oil was re-dissolved in dry MeOH (10 mL) and cooled to 0 °C. Et_3N (1.4 mL, 9.9 mmol) was added dropwise to the solution whilst iodine (1.1 g, 4.3 mmol) was crushed and dissolved in dry MeOH (20 mL). The iodine solution was then added dropwise to the reaction solution until the colour stayed dark brown. After 1 h of stirring at 0 °C, EtOAc (10 mL) was added to the reaction mixture. The solution was washed with 1M HCl (10 mL), 10% $Na_2S_2O_3$ (10 mL) and brine (10 mL). The organic solution was dried over $MgSO_4$ and concentrated under reduced pressure to yield the product **6** as a pale yellow oil (940 mg, 3.00 mmol, 94%).

^{1}H NMR (400 MHz, $CDCl_3$) δ 4.99 (1H, d, *J* = 8.0 Hz, NH), 4.18-4.08 (1H, m, C-2), 1.74-1.65 (1H, m, C-3), 1.52-1.40 (2H, m, C-4), 1.37-1.27 (1H, m, C-3), 1.49 (9H, s, ^{t}Bu), 1.44 (9H, s, ^{t}Bu), 1.01 (3H, s, C-6); **HRMS** (ES+) m/z calculated for $C_{15}H_{27}N_3O_4Na$ $[M+Na]^+$ 336.1894, found 336.1895; $C_{15}H_{27}N_3O_4K$ $[M+K]^+$ 352.1633, found 352.1632; $C_{30}H_{54}N_6O_8Na$ $[2M+Na]^+$ 649.3895, found 649.3893; **R_f** 0.48 (20% ethyl acid in hexane).[2, 4]

Fmoc-photo-Met, 7

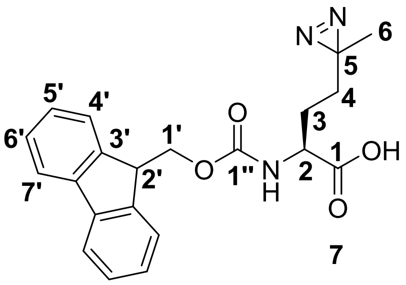

7

4M HCl (25 mL) was added to (S)-tert-butyl 2-((tert-butoxycarbonyl)amino)-4-(3-methyl-3H-diazirin-3-yl)butanoate (250 mg, 0.798 mmol) in THF (25 mL) and the solution was stirred at room temperature until the starting material was consumed. The solvent was removed under reduced pressure and the resulting yellow powder re-dissolved in water/dioxane (1:2) (12 mL). $NaHCO_3$ was added to the solution until the pH was basic. FmocOSu (324 mg, 0.960 mmol) was dissolved in dioxane (1 mL) and added dropwise to the reaction, which was then stirred at room temperature for 24 h. The dioxane was removed under reduced pressure and water (5mL) added to the residue. 1M HCl was added until the pH reached 4 and following this the organic solution was extracted with EtOAc (3 × 10 mL). The combined organics were dried over Na_2SO_4 and concentrated to give the crude product. Purification

using column chromatography (20% EtOAc in hexane, 1% TFA) furnished the product **7** as a white solid (220 mg, 0.580 mmol, 73%).

^{1}H NMR (400 MHz, $CDCl_3$) δ 7.77 (2H, d, *J* = 7.5 Hz, C-4'), 7.58 (2H, d, *J* = 7.5 Hz, C-7'), 7.41 (2H, t, *J* = 7.5 Hz, C-5'/C-6'), 7.32 (2H, t, *J* = 7.5 Hz, C-5'/C-6'), 5.21 (1H, d, *J* = 8.0 Hz, NH), 4.60-4.49 (1H, m, C-2), 4.44 (1H, m, C-1'), 4.40-4.32 (1H, m, C-1'), 4.22 (1H, t, *J* = 6.5 Hz, C-2'), 1.88-1.76 (1H, m, C-3), 1.64-1.34 (3H, m, C-3, C-4); **HRMS** (ES+) m/z calculated for $C_{21}H_{22}N_3O_4$ $[M+H]^+$ 380.1605, found 380.1608; $C_{21}H_{21}N_3NaO_4$ $[M+Na]^+$ 402.1424, found 402.1419; **R_f** 0.34 (60% ethyl acetate in hexane).[2, 5]

1.3 Synthesis of Tracer-FITC

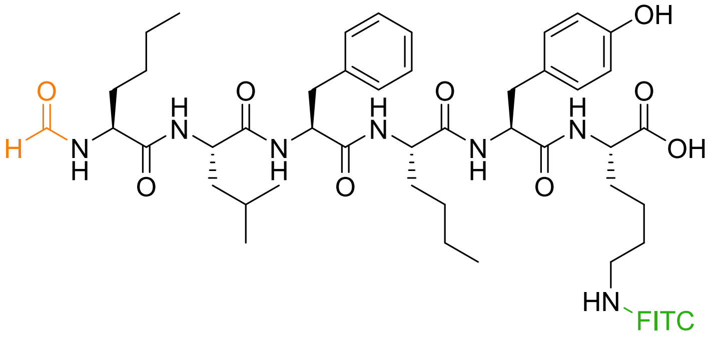

2-Chlorotrityl chloride resin was swollen in DCM for 30 min. Fmoc-Lys(Dde)-OH (1 equiv.) and DIPEA (4 equiv.) were dissolved in dry DCM (2 mL). This solution was added to the swollen resin and rotated for 2 h. The resin was then washed with DCM/MeOH/DIPEA (17:2:1), DCM and DMF sequentially. The lysine residue was deprotected (Fmoc) in 20% v/v piperidine in DMF (2 mL for 3 min × 3) and washed with DMF, DCM and DMF sequentially. Fmoc-Tyr(*t*-Bu)-OH (5 equiv.), Oxyma Pure (5 equiv.) and DIC (5 equiv.) were dissolved in minimal amount of DMF, added to the resin and r for 40 min. The resin was washed sequentially with DMF, DCM and DMF. The remaining amino acids were coupled using the same procedure (Fmoc-Nle, Fmoc-Phe, Fmoc-Leu, Fmoc-NLe). Following Fmoc deprotection of the final amino acid, the N-terminus was formylated: *p*-Nitrophenyl formate (5 equiv.) and DIPEA (10 equiv.) were dissolved in DCM (2 mL) and added to the resin which was subsequently stirred for 12 h. The resin was washed sequentially with DMF, DCM and DMF. The lysine side chain was deprotected (Dde) in 4% v/v hydrazine monohydrate in DMF (2 mL for 3 min × 3) and washed with DMF, DCM and DMF sequentially. FITC (6 equiv.) and DIPEA (10 equiv.) were dissolved in the minimal amount of DMF, added to the resin and rotated for 12 h in darkness. The resin was washed sequentially with DMF, DCM and MeOH, and dried overnight *in vacuo*. The peptide was cleaved from the resin with 2.5% TIS, 2.5% water and 95% TFA and precipitated in cold diethyl ether. The isolated peptide was then purified using UV-directed HPLC. Gradient from 0.1% TFA/ 5% MeCN (v/v) in water to 0.1% TFA/ 95% MeCN (v/v) in water over 15 min. The peptide was obtained from lyophilisation as a yellow amorphous solid in 28% yield.

HRMS (ES+) m/z calculated for $C_{64}H_{77}N_8O_{14}S$ $[M+2H]^{2+}$ 607.2700, found 607.2694.

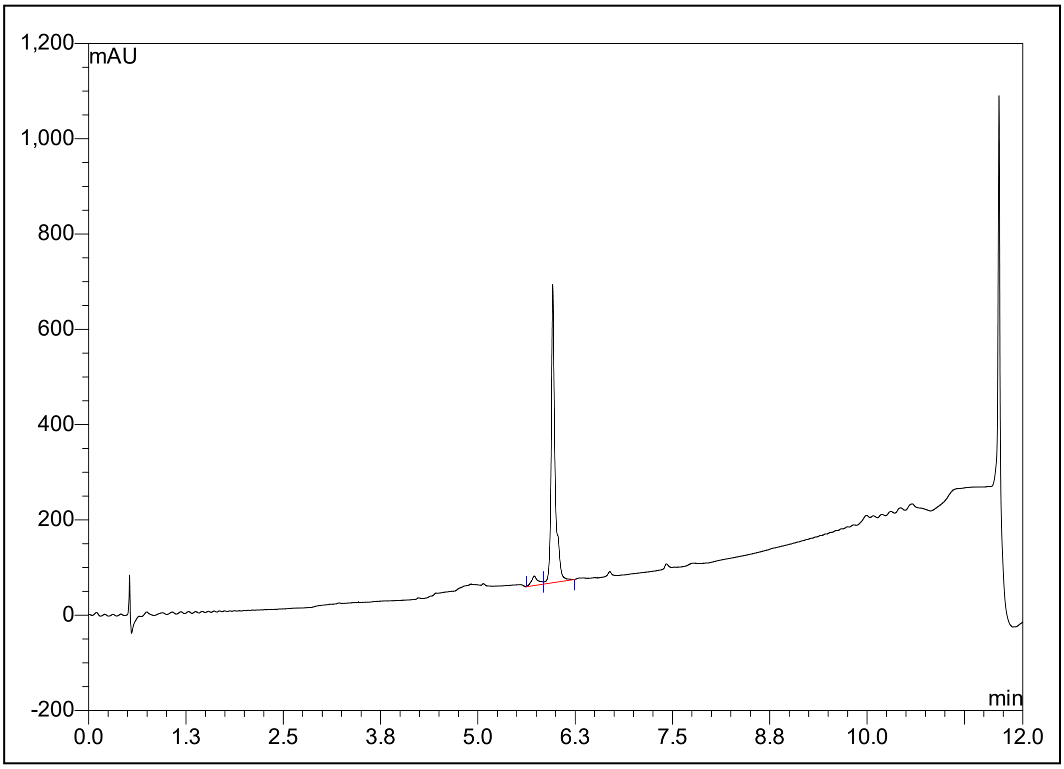

Analytical HPLC trace of purified fluorescent tracer – 95% pure

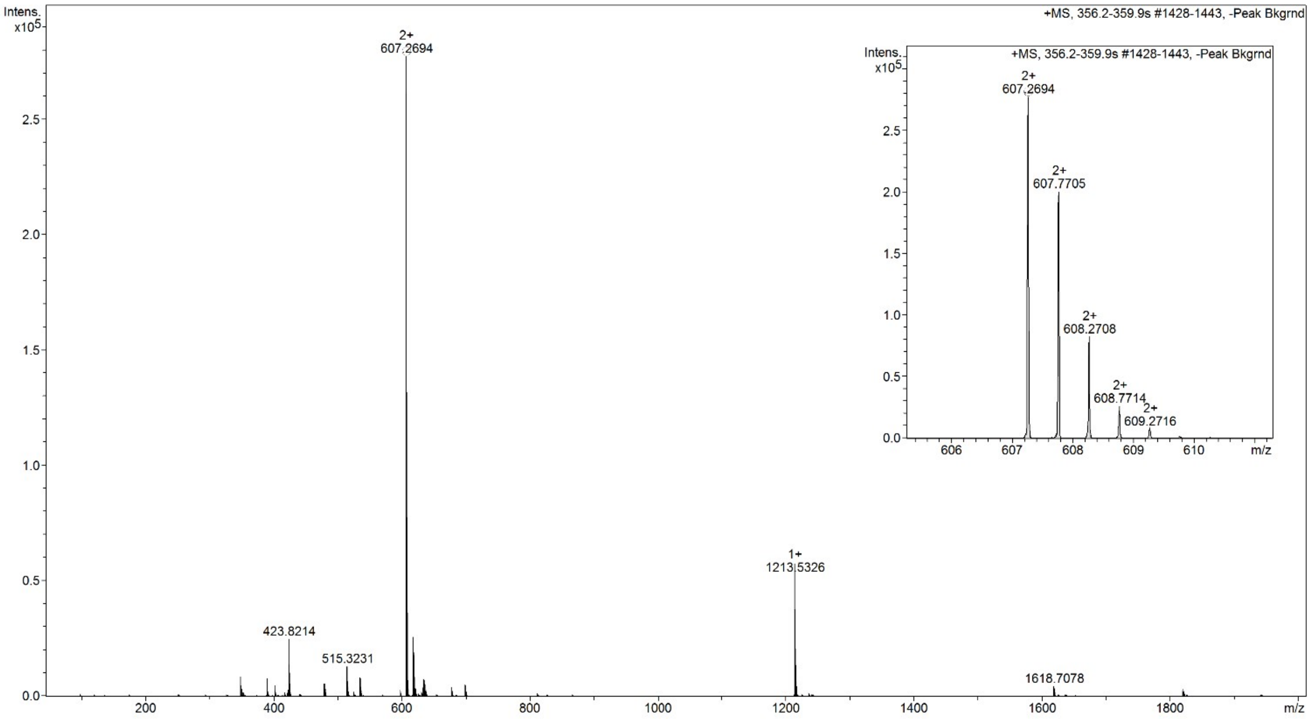

HRMS spectrum of Tracer-FITC. Inset: zoom in on isotope pattern of 607 peak.

1.4 Synthesis of Probe-TAMRA

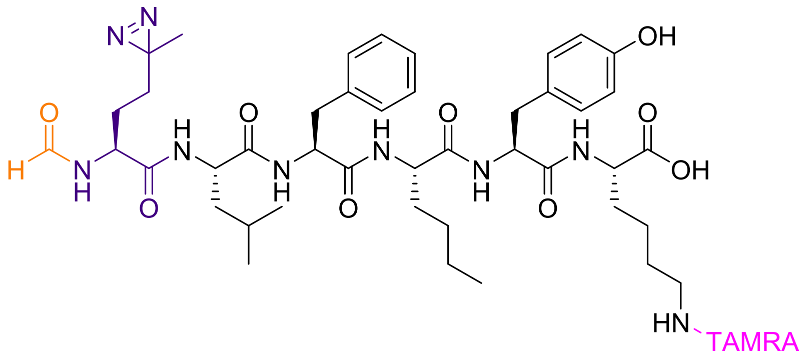

Synthesis and purification as for fluorescent tracer but with Fmoc-photo-Met in place of Fmoc-Nle. 5(6)-TAMRA was coupled to the lysine side chain using Oxyma pure and DIC as with the other amino acids. The peptide was obtained after prep HPLC and lyophilisation as a bright pink amorphous solid in 16% yield.

HRMS (ES+) m/z calculated for $C_{68}H_{83}N_{11}O_{13}$ $[M+2H]^{2+}$ 632.8159, found 632.8170.

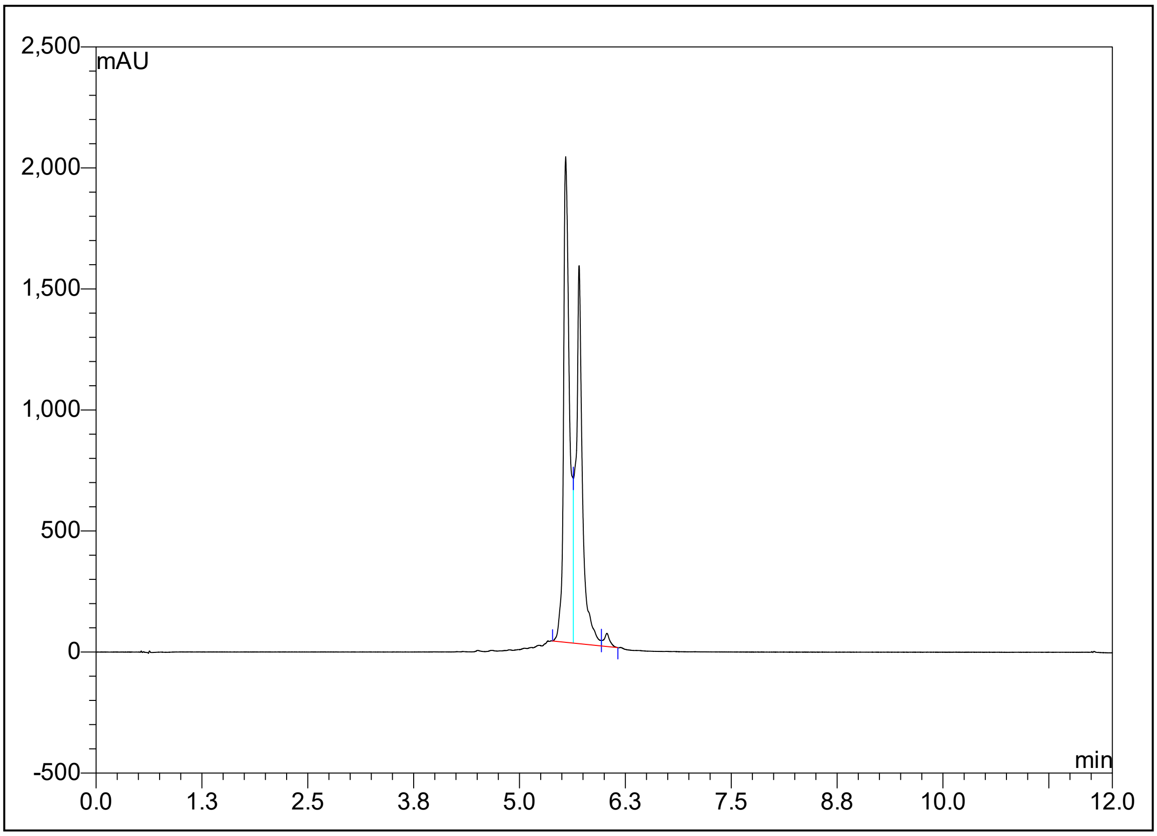

Analytical HPLC trace of purified TAMRA-probe. Two peaks are observed corresponding to the 5- and 6-carboxy isomers. Combined purity 98%

Cmpd 8, 5.4 min

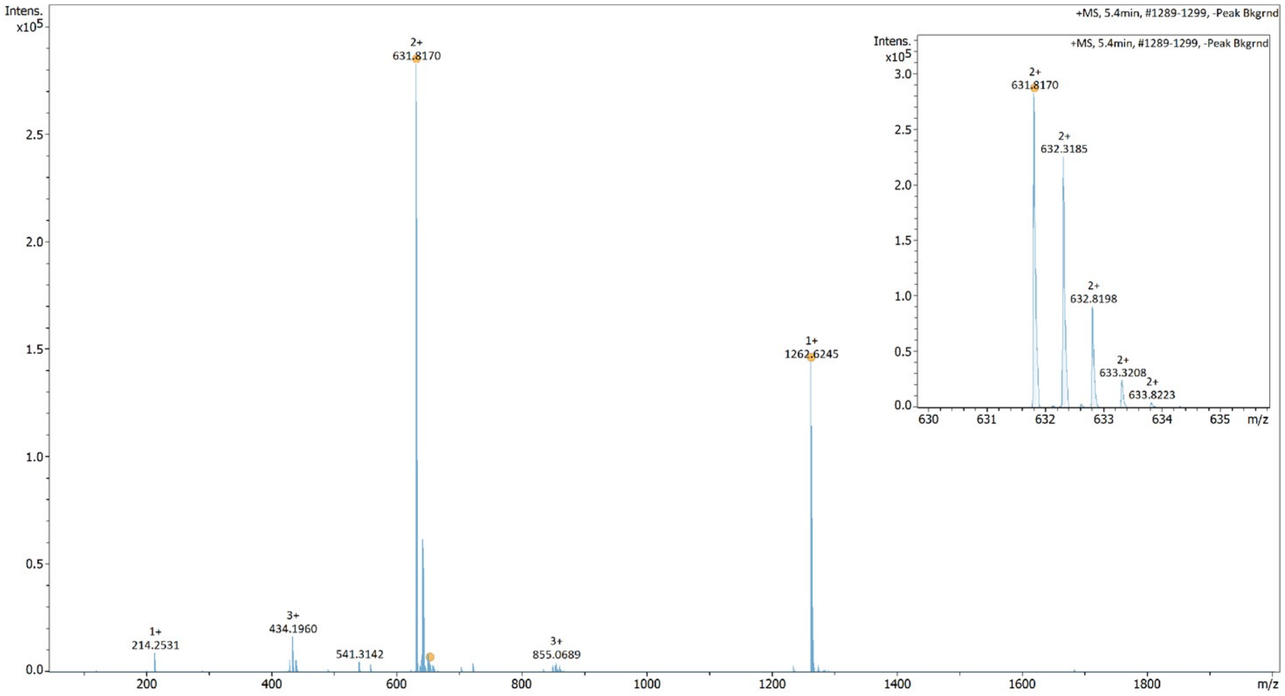

Cmpd 11, 5.5 min

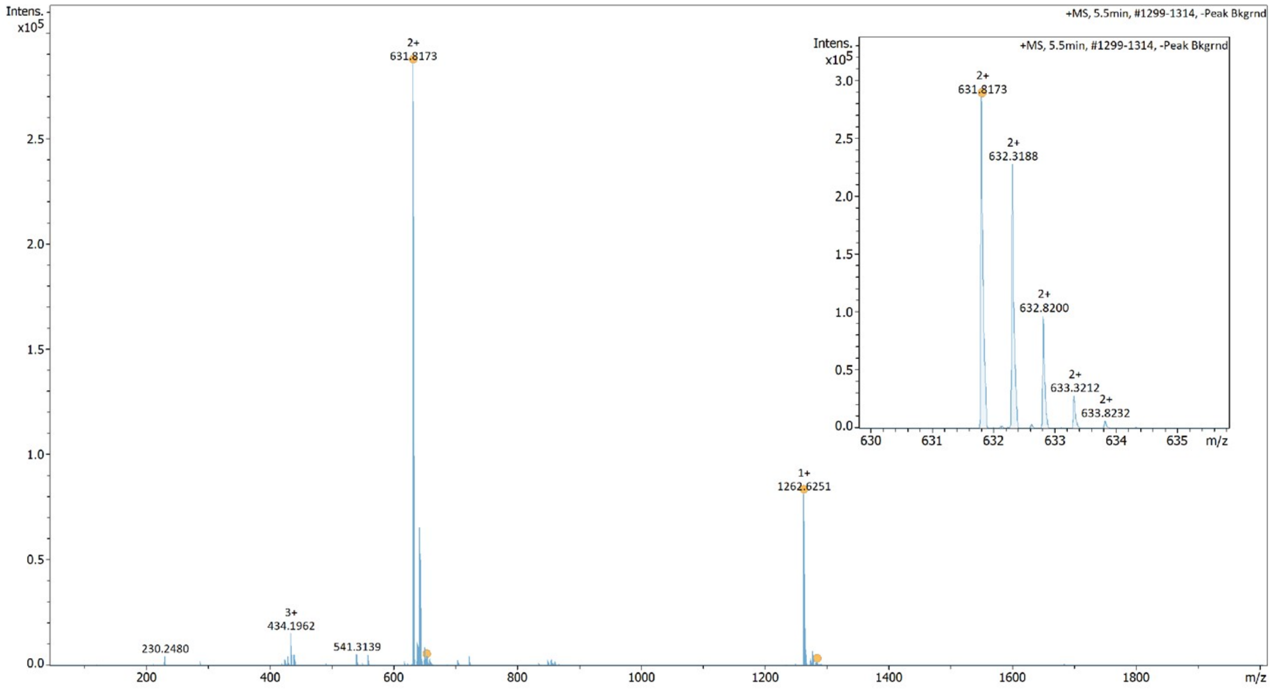

HRMS spectrum of Probe-TAMRA isomers 1 and 2 (top and bottom). Insets: zoom in on isotope pattern of 631 peak.

2 Biological and biochemical methods

2.1 General Methods and Equipment

Reagents were obtained from Sigma-Aldrich, Fisher Scientific and VWR International. All recipe components were dissolved in 18.2 MΩ H_2O to the final volume stated. The pH of the solutions was adjusted using 1 M NaOH or 5 M HCl.

Sterilisation of media, buffers and appropriate equipment was performed using a Prestige Medical bench top autoclave. Thermo Electron Corporation Holten LaminAir laminar flow cabinet was used to maintain a sterile environment when necessary. Bacterial cultures were incubated using a Stuart Orbital Incubator and LB-agar plates were incubated in a Binder BD23 incubator. Centrifugation was performed using either a Heraeus multifuge 3 S-R centrifuge or a Heraeus Fresco-17 centrifuge. Spectrophotometric readings were measured using a Thermo Scientific NanoDrop 2000. SDS-PAGE was carried out using a BioRad Mini-PROTEAN Tetra Cell system and a BioRad Power PAC 1000. A BioRad ChemiDoc MP Imaging System was used to image polyacrylamide gels and Western blots using a combination of UV and white light (DyLight 550 602/50 green epifluorescence, Coomassie Blue 715/30 far red epifluorescence, Chemiluminescent 647SP no light). High resolution ES+ mass spectra were obtained on a Bruker MaXis Impact mass spectrometer.

2.2 Media and buffers

2.2.1 Growth media

LB media: 25 g L^{-1} LB freeze-dried powder (Fisher) in H_2O, sterilised in an autoclave for 20 min at 120 °C.

LB-agar media: 25 g L^{-1} LB freeze-dried powder (Fisher) and 15 g L^{-1} of Agar powder (Fisher) in H_2O, sterilised in an autoclave for 20 min at 120 °C.

2.2.2 Buffers for protein and DNA analysis

Lysis buffer: 0.1% (w/v) SDS, 1% (v/v) Triton X-100, 150 mM NaCl, 50 mM Tris (pH 7.5), 1 × EDTA-free protease inhibitors.

Phosphate-buffered saline (PBS) pH 7.4 (purchased as tablets from Fisher): 0.01 M phosphate buffer, 0.0027 M potassium chloride, 0.137 M NaCl.

SDS-PAGE separating gel buffer: 1.5 M Tris-HCl pH 6.8.

SDS-PAGE stacking gel buffer: 0.5 M Tris-HCl pH 8.8.

SDS-PAGE loading buffer: 62.5 mM Tris-HCl, 5% (w/v) β-mercaptethanol, 10% (w/v) glycerol, 2.5% (w/v) SDS, 0.002% (w/v) bromophenol blue.

SDS-PAGE running buffer (×5): 125 mM Tris-base, 960 mM glycine, 0.5% (w/v) SDS

Western blot transfer buffer: 1.51 g Tris-base, 7.2 g glycine, 100 mL methanol, 400 mL H_2O.

Western blot blocking buffer (3%): 1.50 g fat-free dried milk powder in PBS.

Coomassie stain: Coomassie G-250, 40% (v/v) methanol, 10% (v/v) acetic acid in H_2O.

Coomassie destain: 40% (v/v) methanol, 10% (v/v) acetic acid.

Ponceau Stain: 0.1% w/v Ponceau S in 5% acetic acid.

TBS: 50 mM Tris-HCl, 150 mM NaCl, pH 7.4.

Tris-acetate-EDTA (TAE) (×50): 2 M Tris-HCl, 20 mM acetic acid, 1 mM EDTA.

2.3 Bacterial Transformation of FPR1 gene cDNA ORF

2.3.1 Transformation of *E. coli* Cells

10 µL of *E. coli* competent cells and 2 µg of plasmid containing FPR1 gene (GenScript, OHu10847) were mixed together in a sterile Eppendorf tube on ice. The cells were incubated for 25 min to allow diffusion of the plasmid, followed by a heat shock for 30 s at 42 °C and a further incubation on ice for 5 min to allow uptake of the plasmid. 1 mL of LB media was added to the cells and incubated at 37 °C with shaking (200 rpm) for 1 h. A 100 µL and concentrated aliquot were used to inoculate sterilised agar plates made using ampicillin (100 µg/mL). The plates were incubated overnight at 37 °C and transferred to the fridge in the morning to halt growth.

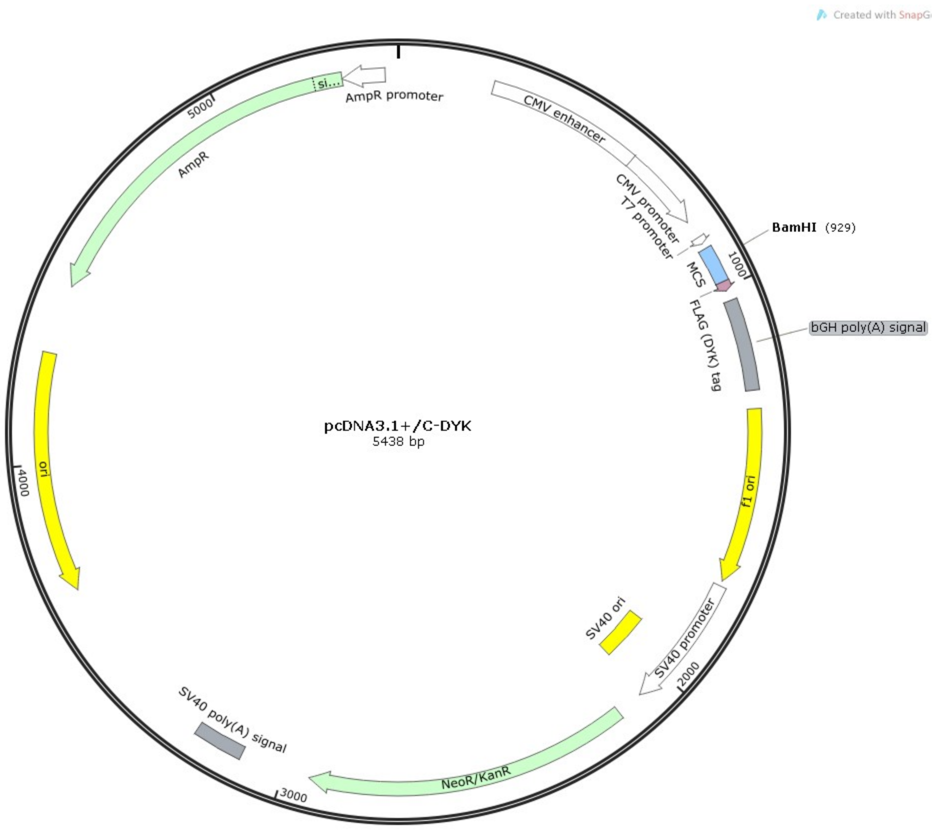

Map of plasmid containing FPR1 gene

2.3.2 Plasmid isolation and quality control

A single colony from the transformed *E. coli* cells was transferred to 35 mL of LB media containing ampicillin (100 µg/mL) and incubated at 37 °C with shaking overnight. A QIAGEN Plasmid Plus Midi Kit was used to obtain the transformed plasmid.

300 ng of plasmid was mixed with 1 unit of BamHI and CutSmart buffer (×1) and made to 50 µL with H_2O. The sample was incubated at 37 °C for 40 min and then stored at –20 °C. Analysis of this digestion was performed with the use of agarose gel electrophoresis using Tris-acetate gels. These were prepared by adding 1% w/v agarose to 40 mL of TAE buffer and heating for 45 s to near boiling. The solution was allowed to cool and 0.4 µL of SYBR stain was added. The solution was poured into a mould with a comb added and allowed to set. The set gel was placed in a gel tank and DNA samples were loaded in loading buffer. Gels were run with TAE buffer (diluted to ×1) at 400 V for 40 min.

2.4 Mammalian cell culture, photocrosslinking and lysis

Cells were incubated in a humidified atmosphere at 37 °C containing 5% CO_2. HEK293T cells were cultured in Dulbecco's Modified Eagle Medium (DMEM, Fisher) supplemented with 1% L-glutamine, 10% v/v fetal bovine serum (FBS) and 1% v/v Pen-Strep. Cells were detached using 1 × trypsin in PBS.

2.4.1 Transient Transfection

HEK293T cells were seeded in a 6-well plate (2 × 10^5 cells/well) and incubated at 37 °C overnight. The plasmid containing FPR1 gene (0.7 µg/µL stock) and Turbofect (Fisher) were mixed with DMEM (no FBS) and incubated at room temperature for 15 min (1 µg of plasmid and 3 µL of Turbofect in 100 µL of media per well). The solution was added to the plated cells and incubated at 37 °C overnight. For mock transfection, the plasmid was omitted.

For 10 cm plate transfection for lysate preparation, the following modifications were used: 1.6×10^6 cells were seeded per plate; 8 µg plasmid and 24 µL Turbofect were mixed in 800 µL DMEM.

2.4.2 Probe Incubation, crosslinking and lysis on suspended cells

Transfected cells were washed with PBS and incubated in 1 mL of PBS with EDTA (per well) at 37 °C for 3 min to detach. The cells were then transferred into sterile Eppendorf tubes and centrifuged at 2000 rpm for 3 min at 4 °C to form pellets. Subsequently the cells were incubated with 1 mL of 10 nM probe in PBS at 0 °C for 30 min. Following incubation the cells were washed with PBS (1 mL × 2) and irradiated with UV light (365 nm) for 30 s using a UV LED device developed for diazirine crosslinking.[6]

Samples were centrifuged at 2000 rpm for 3 min at 4 °C and the PBS solution removed. 200 µL of lysis buffer was added and the lysate chilled for 15 min. Cell debris was pelleted by centrifugation at 13,300 rpm for 15 min at 4 °C. Protein concentration was determined by the DC protein assay (Bio-Rad) using BSA to generate a standard curve. Lysates were kept at –80 °C until required.

2.4.3 Probe incubation and crosslinking experiments on lysates

Transfected cells from 10 cm plates were harvested by scraping in ice-cold PBS and centrifugation at 2000 rpm (500 ×g) for 3 min at 4 °C to form pellets. These were snap frozen and stored at -80 °C until needed. Pellets were thawed, resuspended in 0.5 mL PBS and sonicated 3 × 40% intensity, 10 s, with 30 s on ice in between. Intact cells were removed through centrifugation (500 ×g for 3 min at 4 °C) and the supernatant (total lysate) retained. Crude membrane fractions were prepared by centrifugation (17,000 ×g, 15 min, 4 °C) of the total lysate, removal of the supernatant ("soluble fraction") and resuspension of the pellet ("membrane fraction") in 500 µL PBS. Aliquots were flash frozen.

For competition labelling experiments, 50 µL aliquots of crude membrane fraction were thawed on ice, co-incubated with Probe-TAMRA (50 nM) and various competitors (fMLF, BocMLF or fMLFF at final concentrations of 5 µM, 20 µM, 5 µM respectively) or DMSO for 30 min on ice (final DMSO concentration of 2%). Membranes were pelleted by centrifugation (17,000 ×g, 15 min, 4 °C) and the supernatant discarded. Membranes were resuspended in 25 µL PBS, irradiated for 30 s at 365 nm, deglycosylated as described in section 2.6, and then analysed by SDS-PAGE.

2.5 Gel- and Western blot analysis of samples

Crosslinked cell lysate samples were analysed using gel-based fluorescent imaging. Proteins were separated by SDS-PAGE (180 V, 50 min) with 4% stacking gels and 12% resolving gels on a BioRad Mini-PROTEAN Tetra Cell system with 10 µL All blue standards (BioRad). Fluorescence was measured using a BioRad ChemiDoc MP Imaging System with a Cy3 filter.

For Western blot analysis, samples were separated by SDS-PAGE. A PVDF membrane (BioRad) was soaked in MeOH for 1 min, followed by transfer buffer for 1 min. Two squares of extra thick blot paper

(BioRad) and the protein gel were soaked in transfer buffer for 2 min. The transfer sandwich was then prepared in a Trans-Blot Semi-Dry Transfer Cell (BioRad) in the following order: blot paper, PVDF membrane, gel, blot paper. The transfer was run using a BioRad Power PAC 1000 at 15 V for 30 min.

The membrane was soaked in 3% blocking buffer for 1 h at room temperature. The membrane was then incubated with the primary antibody in 10 mL blocking buffer for 2 h at room temperature, washed with 0.05% Tween in PBS (3 × 5 min), incubated with the secondary antibody in 10 mL blocking buffer for 1 h at room temperature and finally washed with 0.05% Tween in PBS (3 × 5 min).

Primary Antibody	Target Species	Working Dilution
FLAG Tag, mAb, mouse (GenScript, A00187-100)	FLAG Tag	1:10,000
Anti-FPR1/2, clone NFPR1, mouse (Merck, MABF271)	FPR1/2	1:1000
**Secondary Antibody**		
Goat anti-Mouse IgG, HRP (ThermoFisher Scientific, 62-6520)	Mouse	1:10,000

Table S1: Antibodies used for Western blotting

A BioRad Clarity ma Western ECL substrate kit was used with a BioRad ChemiDoc MP Imaging System for chemiluminescence detection. The Western blot was then Ponceau stained for 5 min in staining solution followed by destaining in water.

2.6 Deglycosylation of FPR1

Cell lysate sample adjusted to 1 µg/ µL (50 µL, 50 µg). K_2HPO_4 was added to 0.1 M, SDS was added to 1% and NP40 was added to 1%. The sample was agitated for 30 min at room temperature. 20 units of PNGase F was added to the sample and agitated for 1 h at room temperature. A further 20 units of PNGase F was added to the sample and agitated for 1 h at room temperature. Sample loading buffer was added and the sample stored at –20 °C. Deglycosylation was analysed by Western blot as described above.

2.7 Anti-FLAG Pull-Down

Cell lysate sample adjusted to 1 µg/ µL (200 µL, 200 µg). 50 µL of suspended anti-FLAG M2 magnetic beads (Sigma Aldrich, M8823) were transferred to an Eppendorf and washed three times with TBS, once with 0.1 M glycine (pH 3.5), three times with TBS and once with lysis buffer. The cell lysate sample was added to the beads and incubated overnight with agitation at 4 °C. The supernatant was removed to a new tube and the beads washed three times with lysis buffer. 40 µL of 3X FLAG-peptide (Sigma Aldrich, F4799) in TBS (600 ng/µL) was added to the beads and incubated for 30 min with agitation at 25 °C. The elution mixture was removed to a new tube and the sample analysed by SDS-PAGE and Western blot as above.

2.8 Flow Cytometry

Cells were transfected and incubated with the probe as above (section 2.4). The binding of the probes was observed using a CytoFLEX S 4-laser flow cytometer. The cell samples were loaded in a 1.5 mL Eppendorf and 10,000 events were taken per sample at a flow rate of 30 µL/min. For probes containing a FITC group the 488 nm laser with a 525/40 band pass filter was used, for probes containing a TAMRA

group the 561 laser with a 585/42 band pass filter was used. The data produced from the flow cytometer was then analysed using Kaluza.

To calculate a K_d for Probe-TAMRA binding, FPR1 transfected and mock transfected cells treated with Probe-TAMRA at concentrations of 0, 1, 2, 5, 10, 20, 50, 100 and 200 nM were analysed by flow cytometry as described. This experiment was repeated in biological triplicate. Data were elaborated in Excel and Origin. Background values from mock-transfected samples were subtracted from data values for each concentration. The mean and standard deviation across the three replicates were then calculated for this "specific binding" and data fitted and plotted in Origin using a non-linear logistic fit.

2.9 Confocal Microscopy

Cells were plated on a glass plate and transfected as above (section 2.4.1), then incubated with the probe adhered to plate (35 mm glass-bottom plate). The cells were incubated with 1 mL of 10 nM probe in PBS at 0 °C for 30 min. The sample was then warmed to 37 °C and binding and internalisation of the probes was observed using a Zeiss LSM880 + Airyscan inverted confocal microscope. A DPSS 561 nm laser was used to view the TAMRA fluorescence with an objective of 20x. The data produced was then analysed using Zen.

3 Supplementary Figures

Supplementary Figure S1 Transient transfection of Hek293T cells with FLAG-tagged FPR1.

Supplementary Figure S2 Characterisation of Probe-TAMRA by flow cytometry.

Supplementary Figure S3 Optimisation of crosslinking.

Supplementary Figure S4 Deglycosylation and enrichment of probe-FPR1 adduct.

Supplementary Figure S5 Effect of multiple rounds of irradiation on crosslinking.

Supplementary Figure S6 Full gels and blots for main text figure 4.

Supplementary Figure S7 Probe-TAMRA labelling in lysate compared to on live cells.

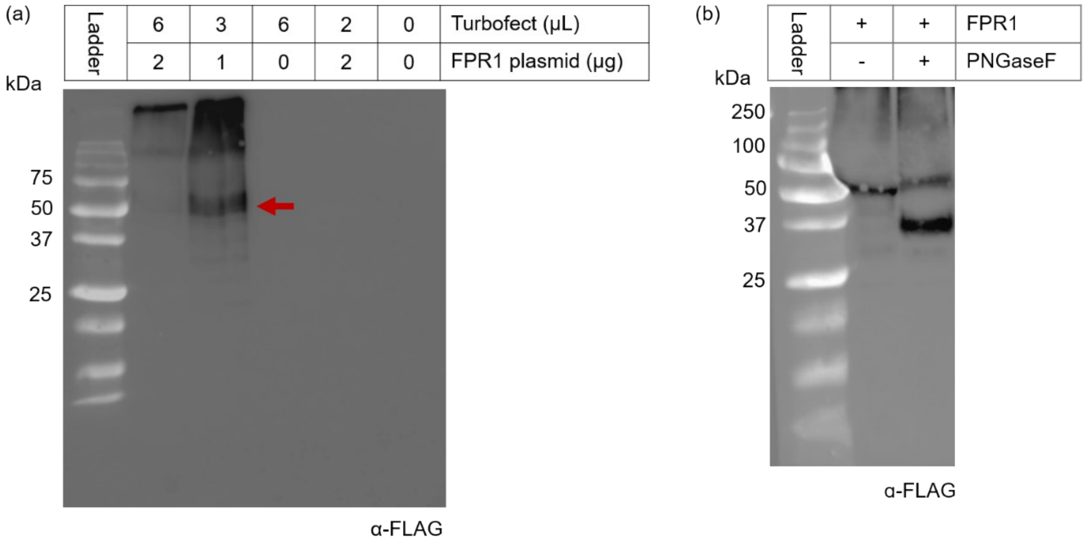

Supplementary Figure S1. Transient transfection of Hek293T cells with FLAG-tagged FPR1 (a) anti-FLAG Wsetern blot showing optimisation of conditions of transient transfection of HEK293T cells with FPR1 plasmid. **(b)** deglycosylation of FPR1 using PNGaseF. Western blot for FLAG tag.

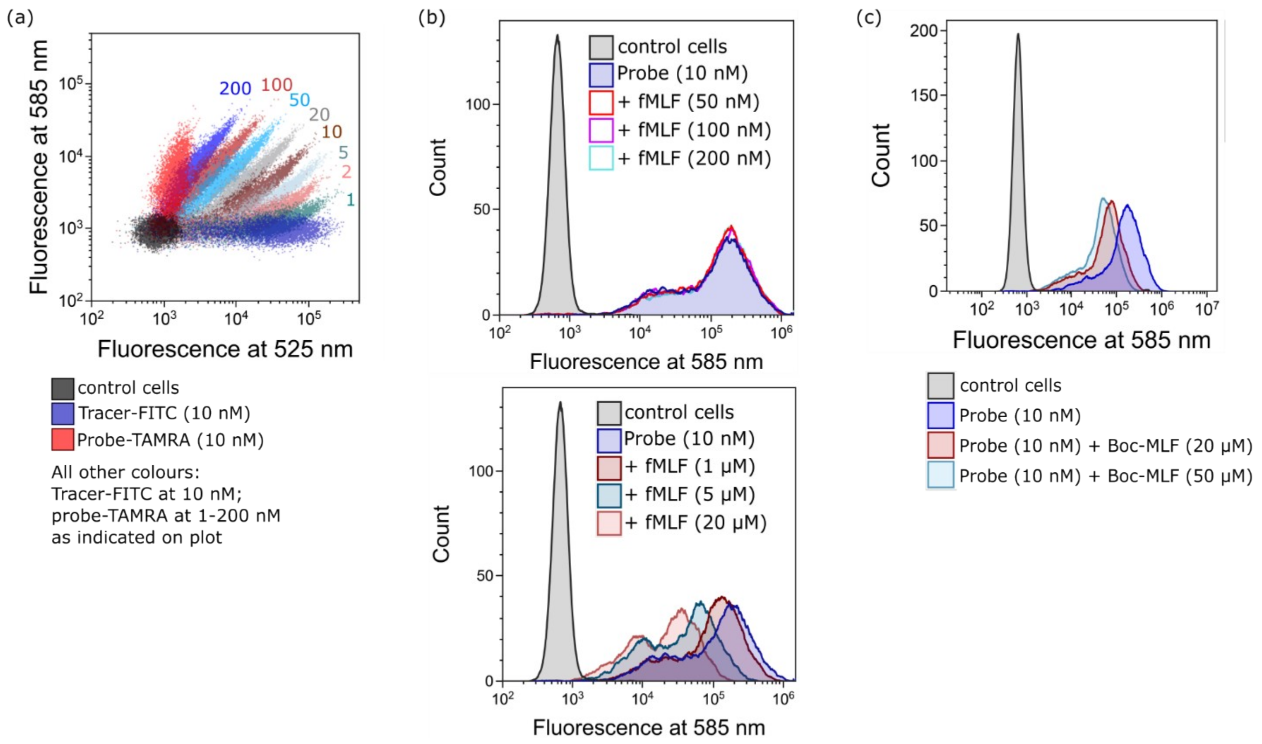

Supplementary Figure S2. Characterisation of Probe-TAMRA by flow cytometry. (a) Dot plot showing competition between Tracer-FITC and Probe-TAMRA; measurements taken at 525 and 585 nm (FITC and TAMRA emissions respectively). Tracer-FITC (10 nM) was incubated with FPR1-HEK293T cells in PBS at 0 °C for 30 min in the presence of increasing concentrations of Probe-TAMRA as indicated. **(b)** Histograms from flow cytometry experiment where probe-TAMRA ("probe") was incubated with FPR1-HEK293T cells at 10 nM in the presence of increasing concentrations of fMLF peptide. Top histogram shows data from experiments using 50-200 nM fMLF and bottom histogram shows data from experiments using 1-20 μM fMLF (separated for clarity). **(c)** Histogram of competition between Probe-TAMRA and Boc-MLF (same conditions as (b)).

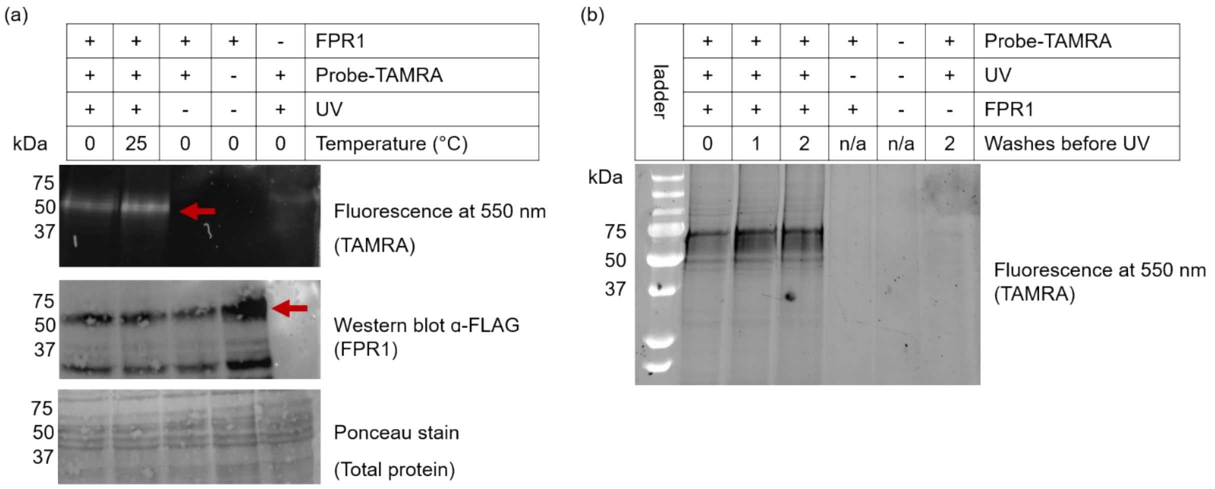

Supplementary Figure S3. Optimisation of crosslinking. **(a) Temperature and UV-dependence of crosslinking.** HEK293T cells transfected with FPR1 or mock transfected (-FPR1) were suspended, incubated with Probe-TAMRA (10 nM) and irradiated with UV light (30 s, 365 nm) on ice (0 °C or at RT) as indicated. Following cell lysis, samples were separated by SDS-PAGE and analysed by in-gel fluorescence (550nm emission) and anti-FLAG Western blot. Ponceau staining was used to assess protein loading. **(b) Effect on crosslinking of washing cells following probe incubation and before irradiation.** Transfected cells were washed 0, 1 or 2 times following probe incubation (10 nM) before UV irradiation and analysis by gel-based fluorescence as above.

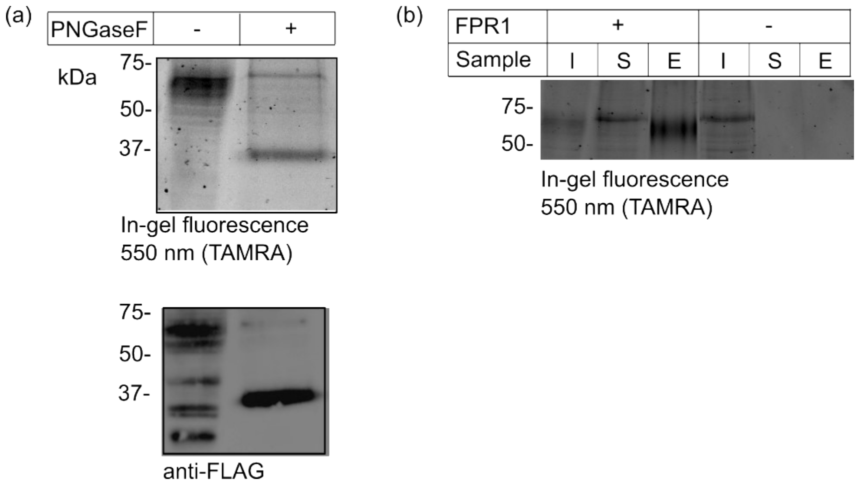

Supplementary Figure S4. **Deglycosylation and enrichment of probe-FPR1 adduct. (a)** Deglycosylation of FPR1-Probe-TAMRA complex following crosslinking, analysed by in-gel fluorescence. Sample taken before and after treatment with PNGaseF. **(b)** In-gel fluorescence analysis of anti-FLAG immunoprecipitation of FPR1-Probe-TAMRA complex following crosslinking. 10 µg loaded for input (I) and supernatant (S); 200 µg pulled down, eluted with FLAG peptide and half of the elution (E) loaded.

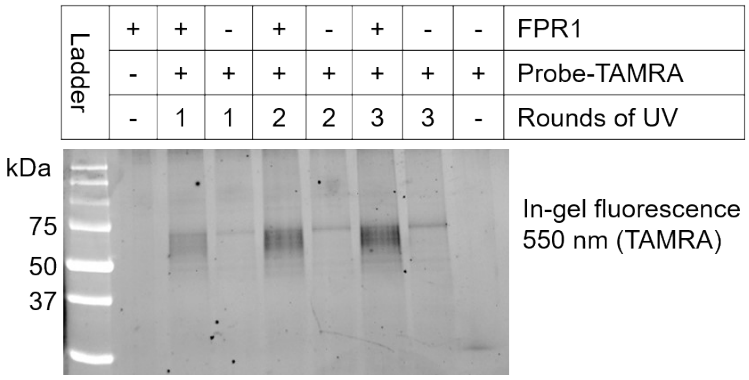

Supplementary Figure S5. Effect of multiple rounds of irradiation on crosslinking. FPR1-transfected HEK293T cells were incubated, as indicated, with Probe-TAMRA (10 nM) and subject to between 1 and 3 rounds of UV irradiation. Each round consisted of probe incubation, UV irradiation (365 nm, 30 s) and washing. The intensities of both FPR1 (~60 kDa diffuse band) and the off-target band at ~75 kDa increase with repeated irradiation steps.

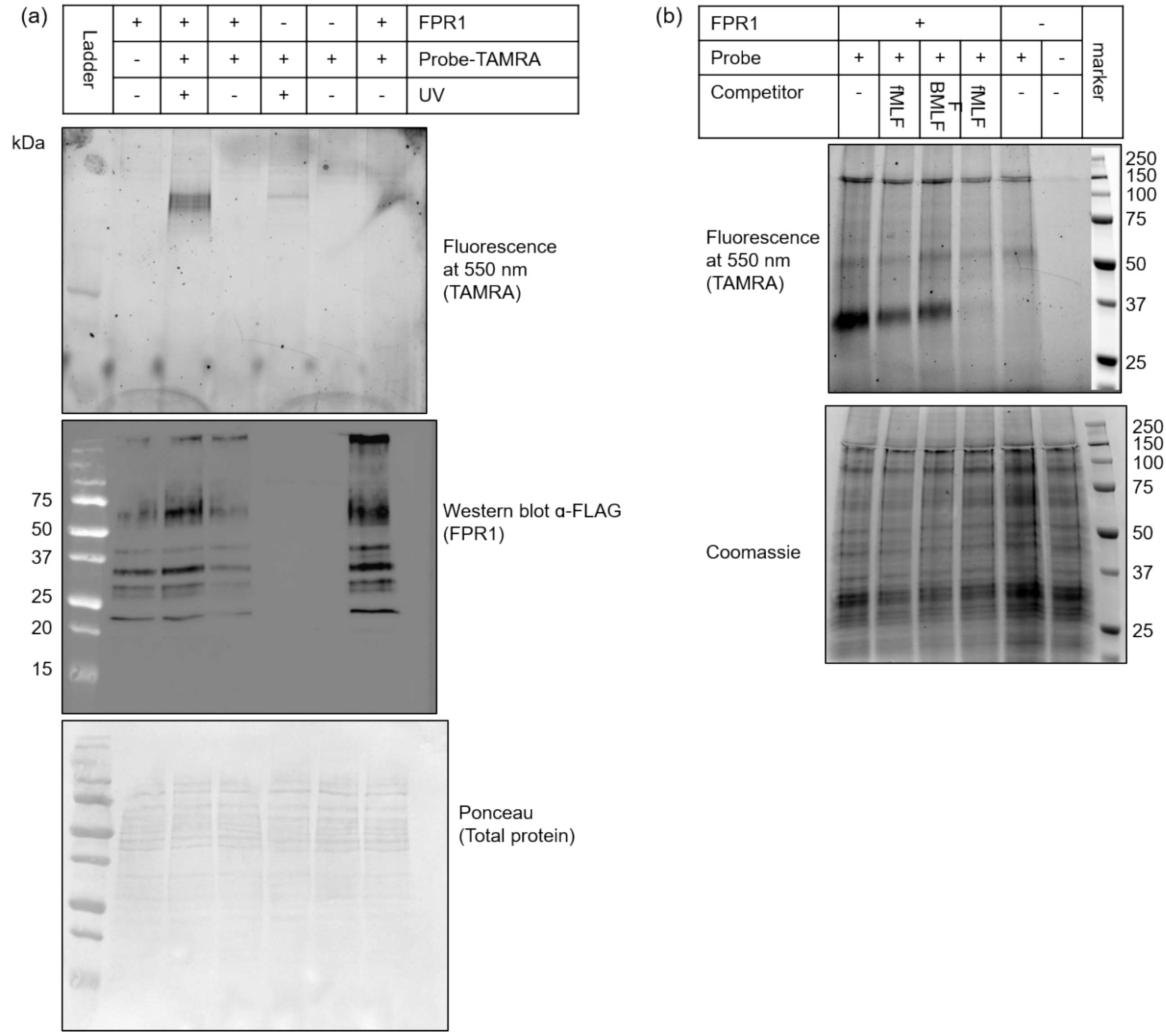

Supplementary Figure S6. Full gels and blots for main text figure 4. (a) UV-dependent crosslinking. HEK293T cells transfected with FPR1 or mock transfected were suspended, incubated with Probe-TAMRA (10 nM) and irradiated with UV light (365 nm) as indicated. Following cell lysis, samples were separated by SDS-PAGE and analysed by in-gel fluorescence (550nm emission) and anti-FLAG Western blot. Ponceau staining was used to assess protein loading. **(b)** Gel-based analysis of competition of Probe-TAMRA labelling with known FPR1 ligands fMLF, BocMLF and fMLFF. HEK293T cells transfected with FPR1 or mock transfected were suspended, lysed by sonication and membrane fraction isolated by centrifugation. Aliquots were co-incubated with Probe-TAMRA (50 nM) and competitor ligand (fMLF: 5 µM; BocMLF: 20 µM; fMLFF: 5 µM) or DMSO control for 30 min, washed, resuspended in PBS and irradiated with UV light (365 nm) for 30 s. Proteins were deglycosylated with PNGaseF, separated by SDS-PAGE and analysed by in-gel fluorescence (550nm emission). The gel was Coomassie stained to provide evidence of equal protein loading.

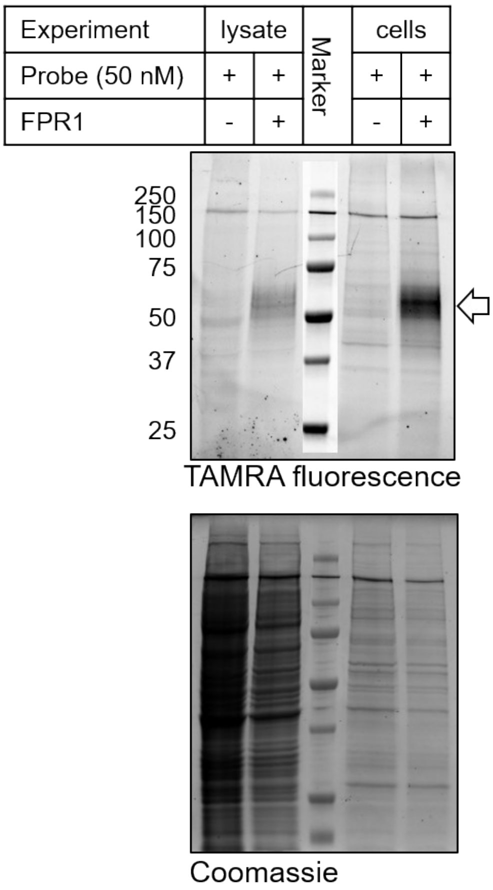

Supplementary Figure S7. **Probe-TAMRA labelling in lysate compared to on live cells.** Cell-based labelling was performed as described previously, with probe concentration increased to 50 nM. For lysate experiments, HEK293T cells transfected with FPR1 or mock transfected were suspended and lysed by sonication in PBS. Aliquots were incubated with Probe-TAMRA (50 nM) for 30 min and irradiated with UV light (365 nm) for 30 s. Proteins were separated by SDS-PAGE and analysed by in-gel fluorescence (550nm emission). FPR1 band is indicated by an arrow The gel was Coomassie stained to evaluate loading.

4 Appendix: NMR spectra for compounds 1-7 (PhotoMet synthesis)

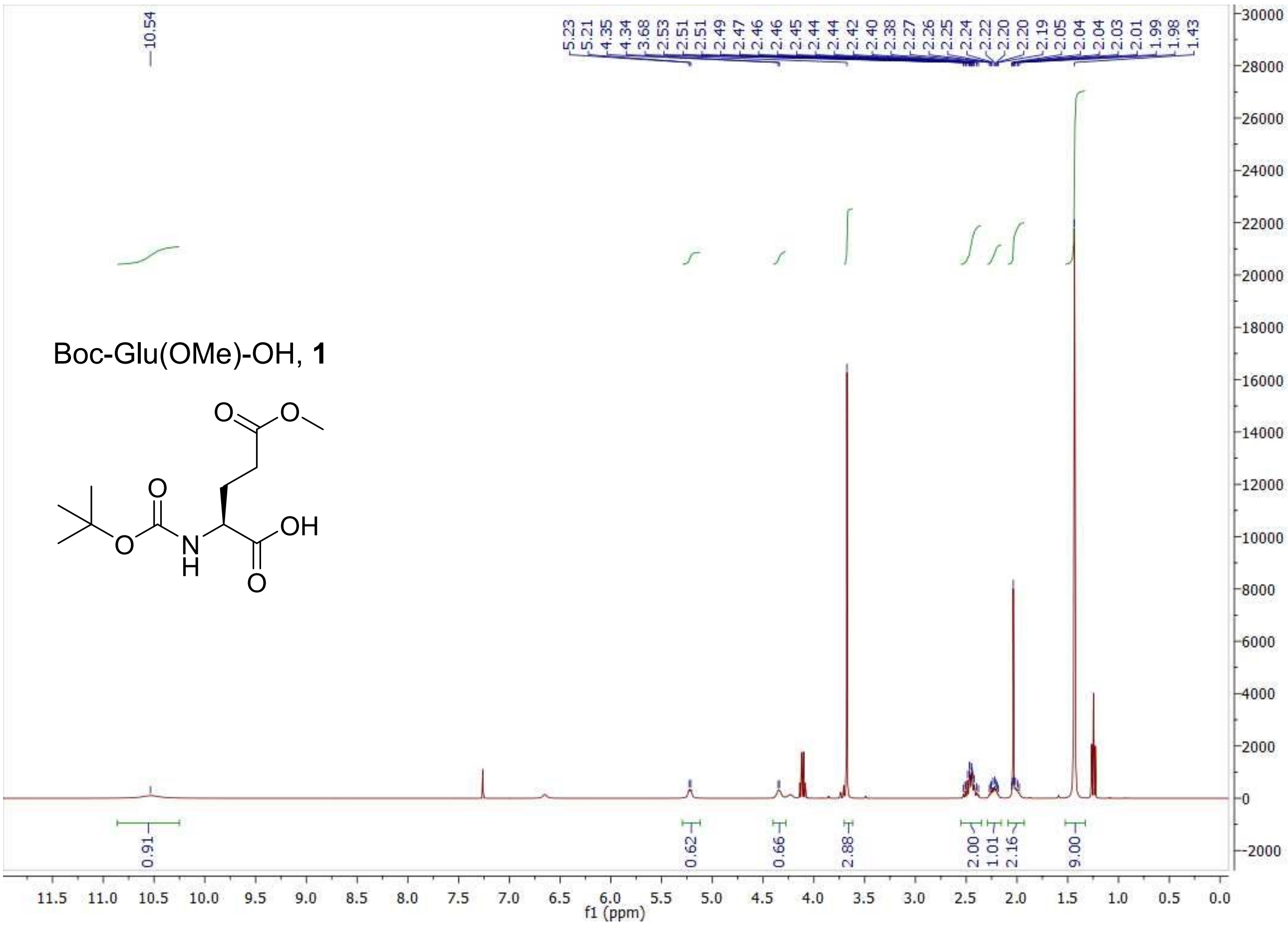

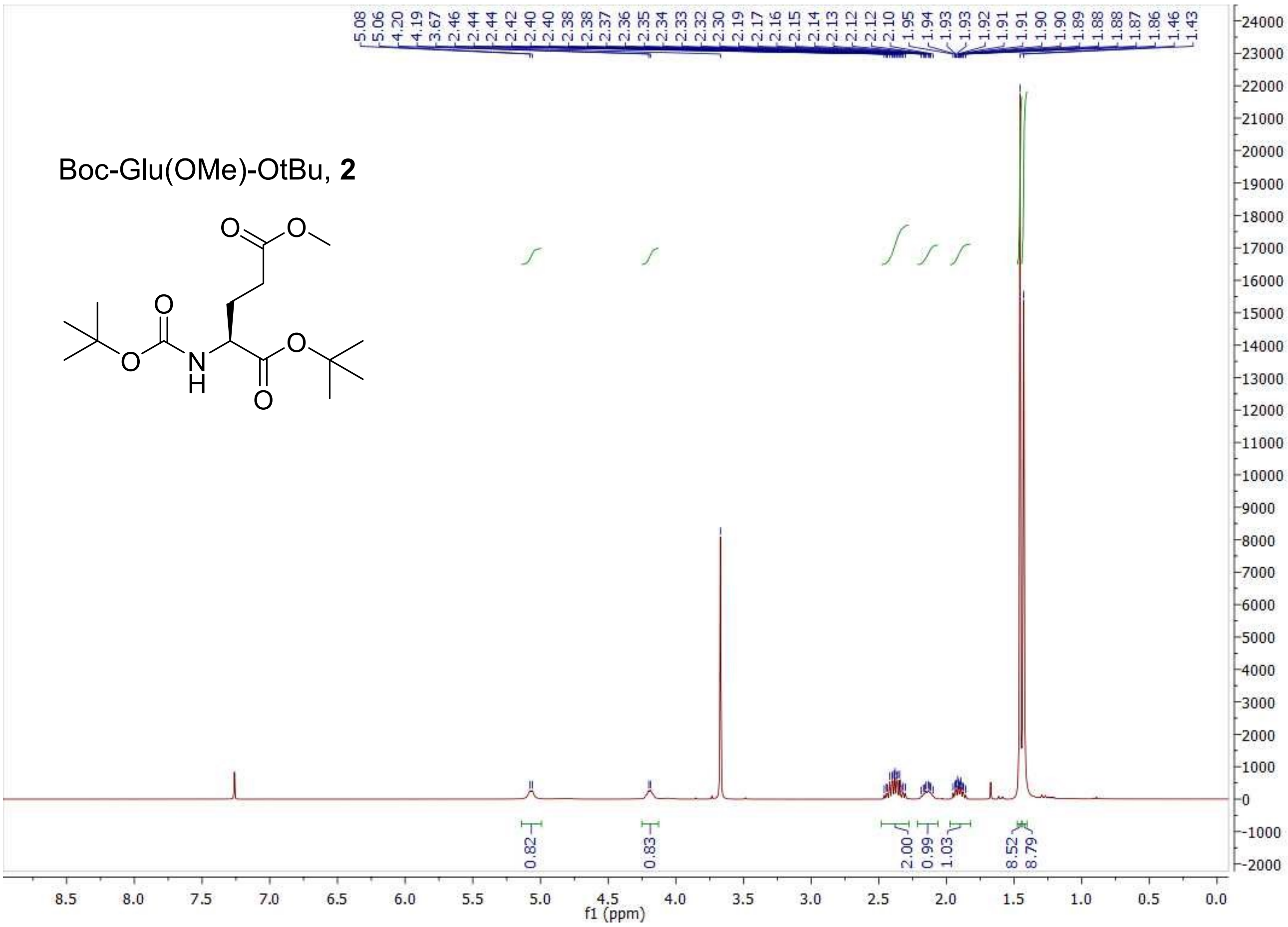

Boc-Glu(OH)-OtBu, **3**

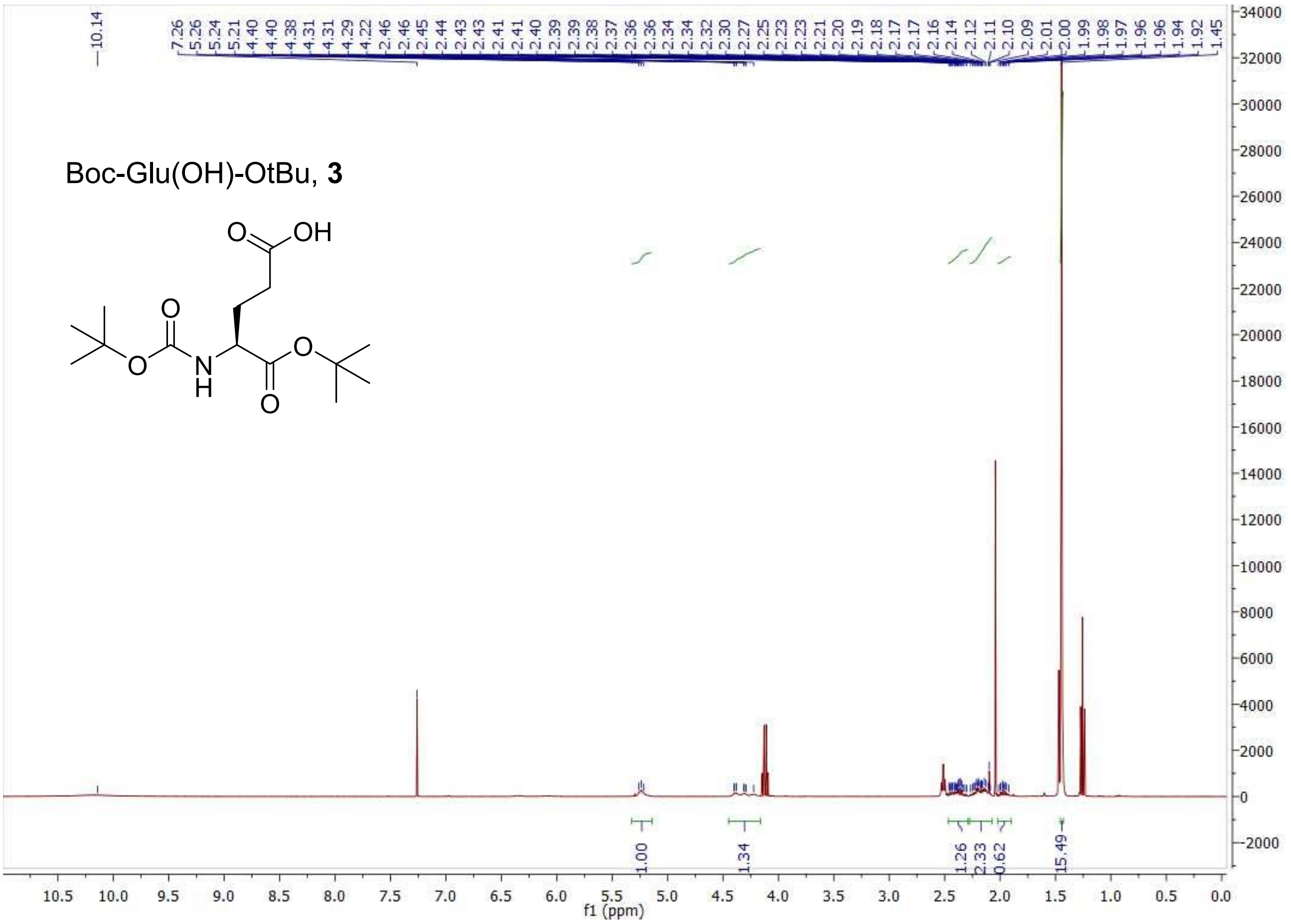

4

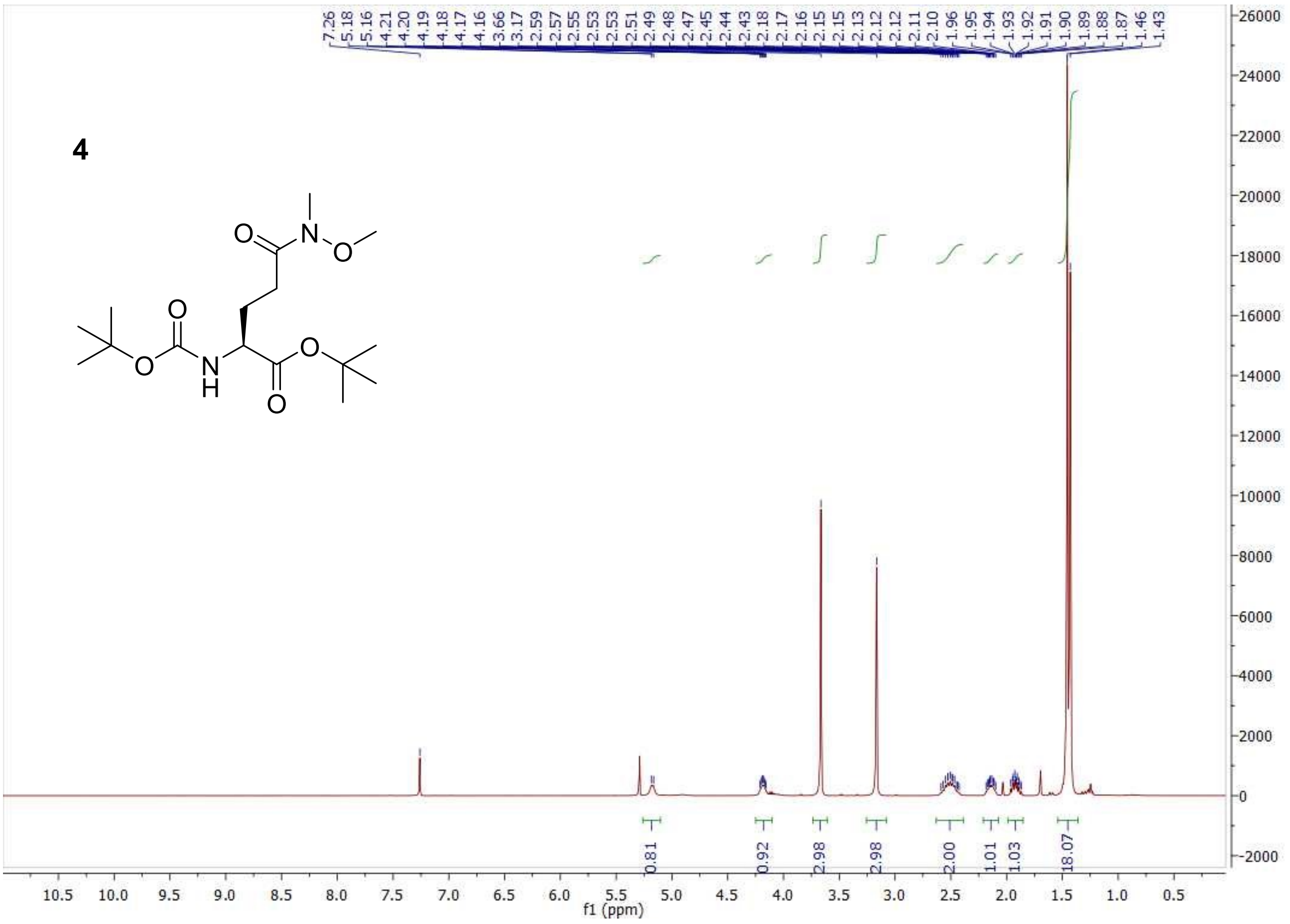

5

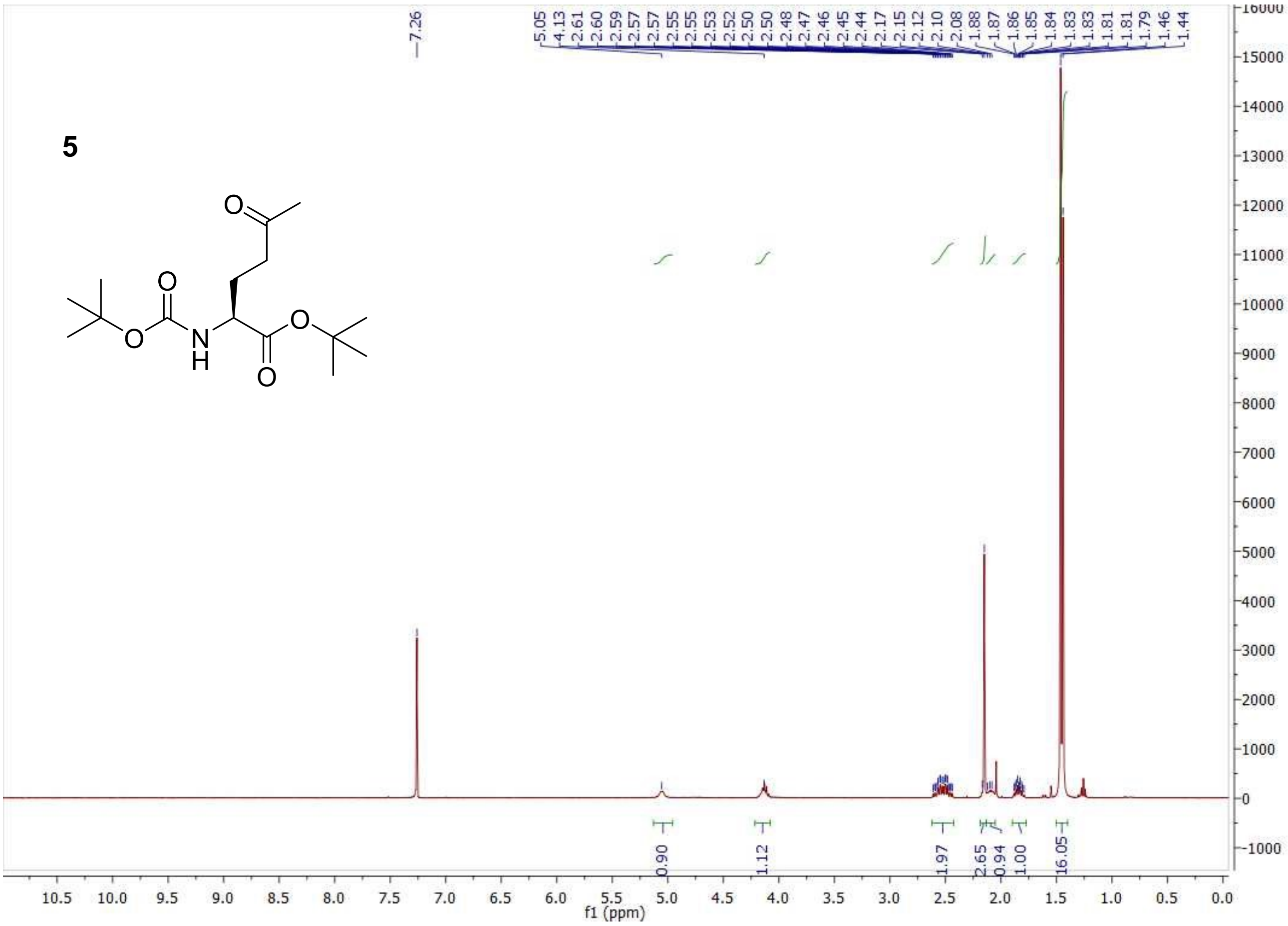

6

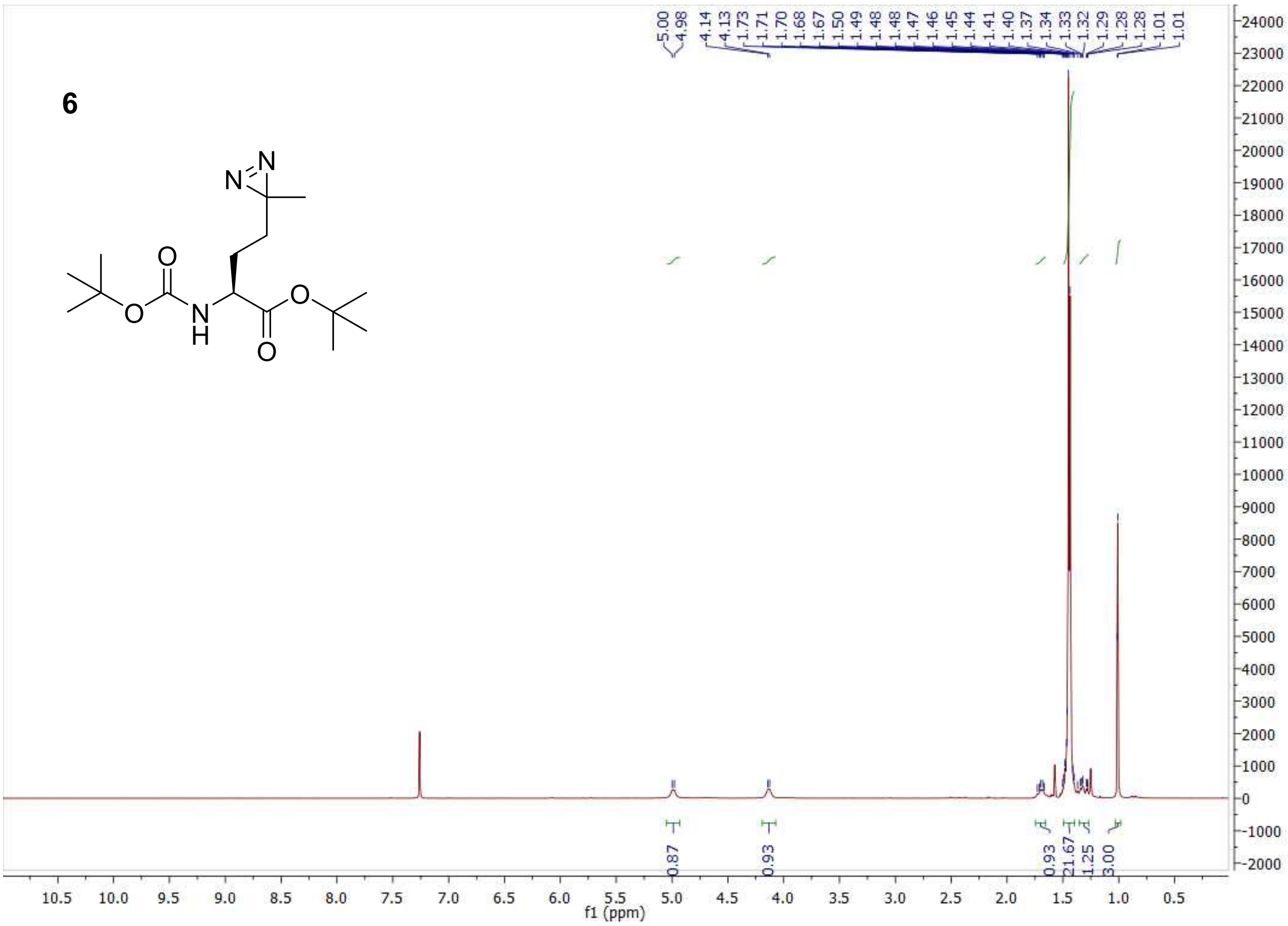

Fmoc-PhotoMet-OH, **6**

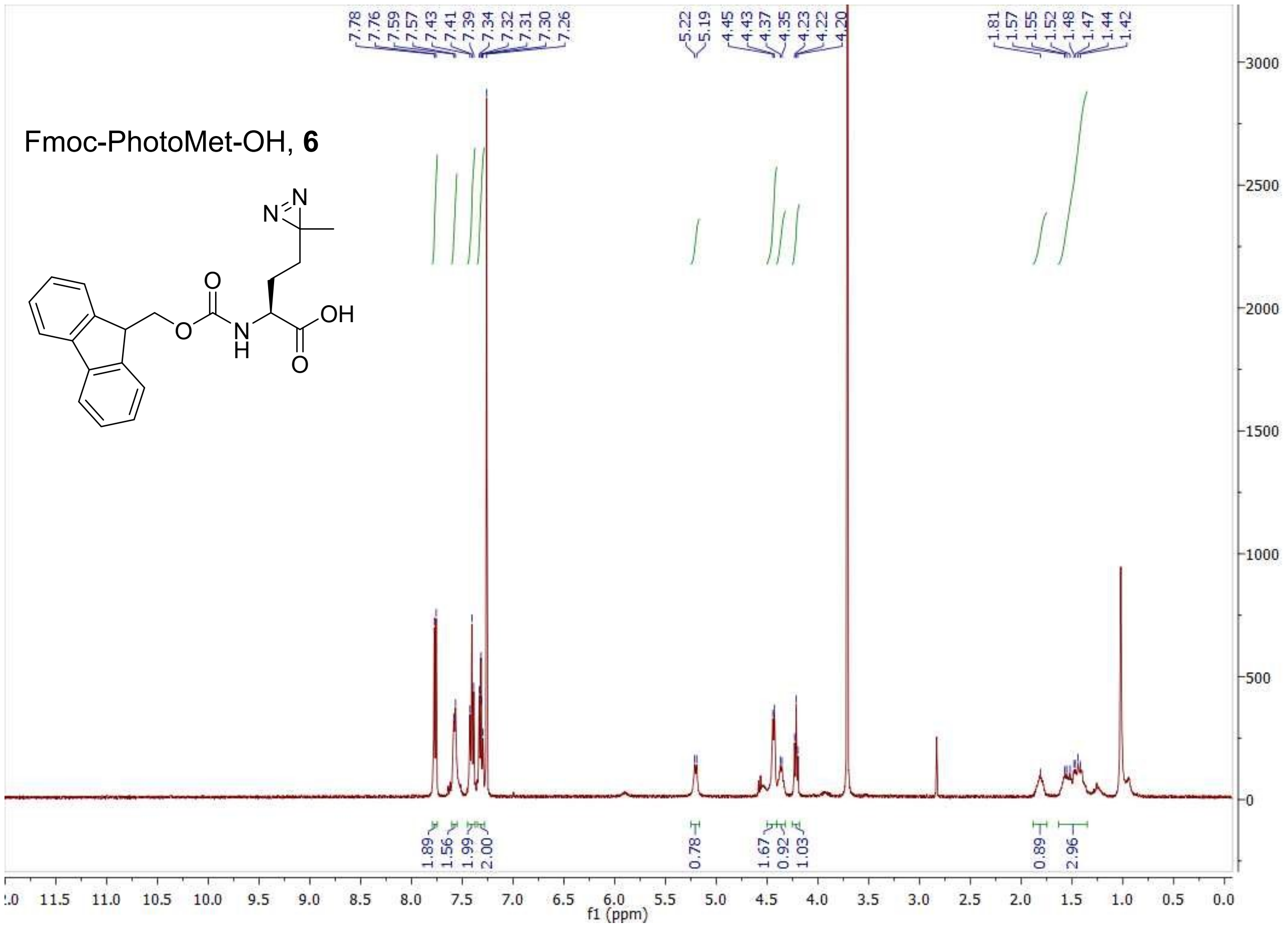

5 References

1. Buckingham, F.; Kirjavainen, A. K.; Forsback, S.; Krzyczmonik, A.; Keller, T.; Newington, I. M.; Glaser, M.; Luthra, S. K.; Solin, O.; Gouverneur, V. r., Organomediated Enantioselective 18F Fluorination for PET Applications. *Angew. Chem. Int. Ed.* **2015,** *54* (45), 13366-13369.
2. Yang, T.; Liu, Z.; Li, X. D., Developing diazirine-based chemical probes to identify histone modification 'readers' and 'erasers'. *Chem. Sci.* **2015,** *6* (2), 1011-1017.
3. Ducho, C.; Hamed, R. B.; Batchelar, E. T.; Sorensen, J. L.; Odell, B.; Schofield, C. J., Synthesis of regio- and stereoselectively deuterium-labelled derivatives of l-glutamate semialdehyde for studies on carbapenem biosynthesis. *Org. Biomol. Chem.* **2009,** *7* (13), 2770-2770.
4. Shi, H.; Zhang, C.-J.; Chen, G. Y. J.; Yao, S. Q., Cell-Based Proteome Profiling of Potential Dasatinib Targets by Use of Affinity-Based Probes. *J. Am. Chem. Soc.* **2012,** *124*, 3001-3014.
5. Vila-Perellö, M.; Pratt, M. R.; Tulin, F.; Muir, T. W., Covalent capture of phospho-dependent protein oligomerization by site-specific incorporation of a diazirine photo-cross-linker. *Journal of the American Chemical Society* **2007,** *129* (26), 8068-8069.
6. Horne, J. E.; Walko, M.; Calabrese, A. N.; Levenstein, M. A.; Brockwelll, D. J.; Kapur, N.; Wilson, A. J.; Radford, S. E., Rapid Mapping of Protein Interactions Using Tag-Transfer Photocrosslinkers. *Angew Chem Int Ed* **2018,** *27*, 16688-16692.
